# Determinants of Antibody Responses to SARS-CoV-2 Vaccines: Population-Based Longitudinal Study (COVIDENCE UK)

**DOI:** 10.3390/vaccines10101601

**Published:** 2022-09-23

**Authors:** David A. Jolliffe, Sian E. Faustini, Hayley Holt, Natalia Perdek, Sheena Maltby, Mohammad Talaei, Matthew Greenig, Giulia Vivaldi, Florence Tydeman, Jane Symons, Gwyneth A. Davies, Ronan A. Lyons, Christopher J. Griffiths, Frank Kee, Aziz Sheikh, Seif O. Shaheen, Alex G. Richter, Adrian R. Martineau

**Affiliations:** 1 Wolfson Institute of Population Health, Barts and The London School of Medicine and Dentistry, Queen Mary University of London, London E1 2AB, UK; 2 Blizard Institute, Barts and the London School of Medicine and Dentistry, Queen Mary University of London, London E1 2AT, UK; 3 Institute of Immunology and Immunotherapy, College of Medical and Dental Sciences, University of Birmingham, Birmingham B15 2TT, UK; 4 Asthma UK Centre for Applied Research, Queen Mary University of London, London E1 2AB, UK; 5 Jane Symons Media, London, UK; 6 Population Data Science, Swansea University Medical School, Singleton Park, Swansea SA2 8PP, UK; 7 Centre for Public Health Research (NI), Queen’s University Belfast, Belfast BT12 6BA, UK; 8 Usher Institute, University of Edinburgh, Edinburgh EH16 4UX, UK

**Keywords:** SARS-CoV-2 vaccines, antibody responses, serology, immunology, epidemiology

## Abstract

Antibody responses to SARS-CoV-2 vaccines vary for reasons that remain poorly understood. A range of sociodemographic, behavioural, clinical, pharmacologic and nutritional factors could explain these differences. To investigate this hypothesis, we tested for presence of combined IgG, IgA and IgM (IgGAM) anti-Spike antibodies before and after 2 doses of ChAdOx1 nCoV-19 (ChAdOx1, AstraZeneca) or BNT162b2 (Pfizer-BioNTech) in UK adults participating in a population-based longitudinal study who received their first dose of vaccine between December 2020 and July 2021. Information on sixty-six potential sociodemographic, behavioural, clinical, pharmacologic and nutritional determinants of serological response to vaccination was captured using serial online questionnaires. We used logistic regression to estimate multivariable-adjusted odds ratios (aORs) for associations between independent variables and risk of seronegativity following two vaccine doses. Additionally, percentage differences in antibody titres between groups were estimated in the sub-set of participants who were seropositive post-vaccination using linear regression. Anti-spike antibodies were undetectable in 378/9101 (4.2%) participants at a median of 8.6 weeks post second vaccine dose. Increased risk of post-vaccination seronegativity associated with administration of ChAdOx1 vs. BNT162b2 (adjusted odds ratio (aOR) 6.6, 95% CI 4.2–10.4), shorter interval between vaccine doses (aOR 1.6, 1.2–2.1, 6–10 vs. >10 weeks), poor vs. excellent general health (aOR 3.1, 1.4–7.0), immunodeficiency (aOR 6.5, 2.5–16.6) and immunosuppressant use (aOR 3.7, 2.4–5.7). Odds of seronegativity were lower for participants who were SARS-CoV-2 seropositive pre-vaccination (aOR 0.2, 0.0–0.6) and for those taking vitamin D supplements (aOR 0.7, 0.5–0.9). Serologic responses to vaccination did not associate with time of day of vaccine administration, lifestyle factors including tobacco smoking, alcohol intake and sleep, or use of anti-pyretics for management of reactive symptoms after vaccination. In a sub-set of 8727 individuals who were seropositive post-vaccination, lower antibody titres associated with administration of ChAdOx1 vs. BNT162b2 (43.4% lower, 41.8–44.8), longer duration between second vaccine dose and sampling (12.7% lower, 8.2–16.9, for 9–16 weeks vs. 2–4 weeks), shorter interval between vaccine doses (10.4% lower, 3.7–16.7, for <6 weeks vs. >10 weeks), receiving a second vaccine dose in October–December vs. April–June (47.7% lower, 11.4–69.1), older age (3.3% lower per 10-year increase in age, 2.1–4.6), and hypertension (4.1% lower, 1.1–6.9). Higher antibody titres associated with South Asian ethnicity (16.2% higher, 3.0–31.1, vs. White ethnicity) or Mixed/Multiple/Other ethnicity (11.8% higher, 2.9–21.6, vs. White ethnicity), higher body mass index (BMI; 2.9% higher, 0.2–5.7, for BMI 25–30 vs. <25 kg/m^2^) and pre-vaccination seropositivity for SARS-CoV-2 (105.1% higher, 94.1–116.6, for those seropositive and experienced COVID-19 symptoms vs. those who were seronegative pre-vaccination). In conclusion, we identify multiple determinants of antibody responses to SARS-CoV-2 vaccines, many of which are modifiable.

## 1. Introduction

Vaccination against SARS-CoV-2 represents a key tool for COVID-19 control. However, existing vaccines offer imperfect protection against disease, reflecting heterogeneity in immunogenicity [1,2]. Improved understanding of factors influencing these responses to SARS-CoV-2 vaccines could lead to discovery of effect-modifiers that could be harnessed to augment vaccine immunogenicity, and identify groups of poor responders who might benefit from more intensive vaccine dosing regimens or implementation of other protective measures [3].

Existing studies investigating determinants of vaccine immunogenicity have reported that lower antibody responses following SARS-CoV-2 vaccination associate with administration of viral vector vs. messenger RNA (mRNA) vaccines, older age, poorer general health, immunosuppression and shorter inter-dose intervals [4,5,6,7,8,9,10,11]. Higher post-vaccination antibody titres are seen in those with evidence of SARS-CoV-2 infection before vaccination [9,12,13]. However, these studies are limited in several respects: many are conducted in specific populations such as health care workers [5,9,12,13] or in individuals with a particular demographic or clinical characteristic that may influence vaccine immunogenicity [6,10,11], which may constrain generalisability of their results. Where population-based studies have been done [4,7,8], these did not compare the effect of two doses of viral vector vs. mRNA vaccines on host response; neither did they systematically ascertain participants’ pre-vaccination SARS-CoV-2 serostatus, which is an important potential confounder of associations reported. Moreover, these studies did not investigate effects of modifiable factors that have been posited to influence responses to vaccination, such as time of day of inoculation [14], nutrition [15], sleep [16], alcohol use [17], tobacco smoking [18] and peri-vaccination use of anti-pyretic analgesics [19].

We sought to address these limitations by investigating a comprehensive range of potential sociodemographic, behavioural, clinical, pharmacologic and nutritional determinants of antibody responses in a population-based cohort of UK adults (COVIDENCE UK) [20] following administration of two doses of either BNT162b2 (Pfizer-BioNTech) or ChAdOx1 nCoV-19 (Oxford-AstraZeneca; hereafter ChAdOx1) SARS-CoV-2 vaccines. We utilised a semi-quantitative assay with proven sensitivity for detection of combined IgG, IgA and IgM antibodies to the SARS-CoV-2 spike antigen [21] that has been validated as a correlate of protection against breakthrough COVID-19 in two populations [22,23].

## 2. Materials and Methods

### 2.1. Study Design and Participants

COVIDENCE UK is a prospective, longitudinal, population-based observational study of COVID-19 in the UK population (www.qmul.ac.uk/covidence accessed on 20 September 2022) [20]. Inclusion criteria were age ≥ 16 years and UK residence at enrolment, with no exclusion criteria. Participants were invited via a national media campaign to complete an online baseline questionnaire to capture information on potential symptoms of COVID-19 experienced since 1 February 2020; results of any COVID-19 tests; and details of a wide range of potential risk factors for COVID-19 and determinants of vaccine response (Table S1, Online Data Supplement). Online monthly follow-up questionnaires captured incident test-confirmed COVID-19 and vaccination details (Table S2, Online Data Supplement), and a pre-vaccination serology study was conducted to evaluate risk factors for SARS-CoV-2 infection [24]. The study was launched on 1 May 2020, and closed to enrolment on 6 October 2021. Participants in the cohort were invited via email to participate in post-vaccination antibody studies and to give additional consent for these. No incentives were offered to participants. The primary analysis presented here includes data from all participants for whom a serology result was available following administration of two doses of ChAdOx1 or BNT162b2.

COVIDENCE UK was sponsored by Queen Mary University of London and approved by Leicester South Research Ethics Committee (ref 20/EM/0117). It is registered with ClinicalTrials.gov (NCT04330599).

### 2.2. Statistical Analysis

Full details of statistical analyses, study procedures, outcomes and sample size calculation are provided in the Online Data Supplement. Briefly, logistic regression models were used to estimate adjusted ORs, 95% (CIs) and associated pairwise *p*-values for potential determinants of post-vaccination seronegativity in all participants who had not reported a SARS-CoV-2 infection prior to their 2nd vaccine dosing date. Linear regression models were used to estimate beta-coefficients, 95% CIs and associated pairwise *p*-values for potential determinants of log-transformed antibody titres in the subset of seropositive participants. For ease of interpretation, log-transformed estimates of antibody titres were exponentiated and a percentage increase or decrease was calculated for every one-unit increase in the potential determinant. We first estimated ORs and beta-coefficients in minimally adjusted models, and included factors independently associated with each outcome at the 10% significance level in fully adjusted models. 

In a sensitivity analysis, we excluded participants from the analysis of antibody titres after two vaccine doses who reported a positive RT-PCR or lateral flow test for SARS-CoV-2 between the date of their second dose of vaccine and the date on which they provided their dried blood spot sample. We also stratified the analysis of antibody titres following two vaccine doses according to the type of vaccine received to explore whether determinants of antibody responses to two vaccine doses were consistent for ChAdOx1 vs. BNT162b2. Given reports that peri-vaccination use of antipyretic analgesics may attenuate vaccine immunogenicity [25], we also conducted an exploratory analysis to determine the influence of taking paracetamol or non-steroidal anti-inflammatory drugs (NSAIDs) to treat post-vaccination symptoms on post-vaccination antibody titres.

## 3. Results

A total of 15,527 participants were invited to participate in the study of antibody responses following two vaccine doses, of whom 13,005 consented to take part. Serology results were available for 9244 participants, of whom 9101 received two doses of ChAdOx1 (*n* = 5770) or BNT162b2 (*n* = 3331) for their primary vaccination course and were included in this analysis (Figure S1, Online Data Supplement). Average participant outcome data completeness was 99.5%. Participant characteristics are presented in Table 1: median age was 64.2 years at enrolment (IQR 57.1, 69.9), 71.1% were female, and 96.5% were of White ethnic origin. Post-vaccination dried blood spot samples were provided from 12 January to 18 September 2021 by participants who received their first dose of vaccine from 15 December 2020 to 10 July 2021 and their second dose from 5 January 2021 to 4 September 2021. Median time from the date of participants’ second vaccine dose to the date on which post-vaccination dried blood spot samples were provided was 8.6 weeks (IQR 6.4–10.7 weeks) and 374 of 9101 participants (4.1%) were seronegative following two doses of vaccine.

### 3.1. Determinants of Seronegativity following a Primary Course of SARS-CoV-2 Vaccination

Table 2 presents results of minimally adjusted and multivariable analyses to identify factors that were independently associated with the absence of detectable anti-spike IgG/A/M following two doses of ChAdOx1 or BNT162b2. After adjusting for age and sex, 32 independent variables associated with post-vaccination serostatus with *p* < 0.10 and were fitted in a fully adjusted model. Nine factors in the fully adjusted model remained independently associated with post-vaccination serostatus (pairwise *p*-value below the multiple comparison test (MCT) threshold of 0.024, and/or *p* for trend <0.05). Seven of these were associated with higher risk of post-vaccination seronegativity: receiving ChAdOx1 vs. BNT162b2 vaccine (aOR 6.62, 95% CI 4.21–10.42); shorter interval between first and second vaccine doses (aOR 1.60, 1.20–2.14, for 6–10 weeks vs. >10 weeks, and aOR 2.56, 1.22–5.37, for <6 weeks vs. >10 weeks); receiving the second vaccine dose during the first quarter (Q1), third quarter (Q3) or the final quarter (Q4) of the year (aOR 1.08, 0.61–1.92, for Q1 vs. Q2, aOR 12.64, 2.21–72.27, for Q3 vs. Q2, and aOR 21.83, 1.74–273.33, for Q4 vs. Q2; older age (aOR per 10-year increase in age 1.68, 1.42–2.00), poorer self-assessed general health (aOR per level of declining health, 1.26, 1.09–1.45); immunodeficiency disorder (aOR 6.48, 2.53–16.59), and use of systemic immunosuppressants (aOR 3.71, 2.41–5.69). Two factors were associated with lower risk of post-vaccination seronegativity: pre-vaccination seropositivity for SARS-CoV-2 (aOR 0.53, 0.32–0.89, for individuals who were seropositive without COVID-19 symptoms pre-vaccination vs. those who were seronegative pre-vaccination, and aOR 0.16, 0.04–0.56, for individuals who were seropositive with COVID-19 symptoms pre-vaccination vs. those who were seronegative pre-vaccination) and regular consumption of vitamin D supplements (aOR 0.66, 0.51–0.87). No associations were seen for other independent variables investigated, including time of day of inoculation, nutrition, sleep, alcohol use, tobacco smoking or socioeconomic deprivation.

### 3.2. Determinants of Post-Vaccination Antibody Titres in Subset of Individuals Who Were Seropositive following a Primary Course of SARS-CoV-2 Vaccination

**Figure 1:**
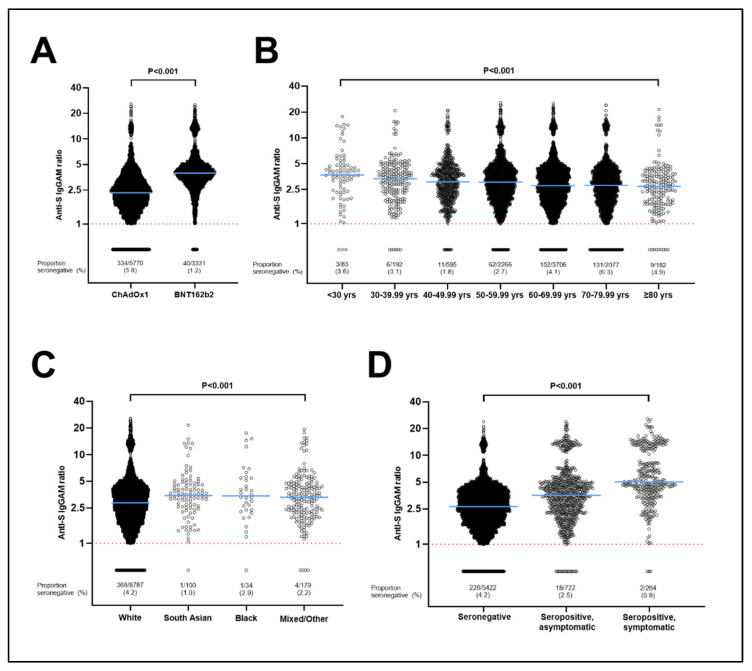
Antibody titres following two doses of vaccine by type of vaccine (**A**), age (**B**), ethnicity (**C**) and pre-vaccination SARS-CoV-2 status (**D**). For C, South Asian indicates people who self-identified their ethnic origin as Indian, Pakistani, or Bangladeshi, and Black indicates people who self-identified their ethnic origin as Black, African, Caribbean or Black British. *p* values from Mann–Whitney test (**A**) and Kruskal–Wallis tests (**B**–**D**). Anti-S, anti-spike; IgGAM, immunoglobulin G, A or M. Dotted line = limit of detection.

To identify factors influencing the magnitude of antibody responses to COVID-19 vaccination, we investigated determinants of SARS-CoV-2 antibody titres in the subset of 8727 participants who were seropositive following their second COVID-19 vaccine dose. Results of this analysis are presented in Table 3. A total of 22 factors associated with antibody titres after adjusting for age and sex with *p* < 0.10 and were fitted in a fully adjusted model. Eight factors remained independently associated with antibody titres in the fully adjusted model (pairwise *p*-values below the MCT threshold of 0.025, and/or P for trend < 0.05). Six of these independently associated with lower antibody titres: receipt of ChAdOx1 vs. BNT162b2 vaccine (43.4% lower titres, 95% CI 41.8–44.8, Figure 1); longer duration between date of second vaccine dose and date of sampling (7.4% lower, 3.0–11.6, for 5–8 weeks vs. 2–4 weeks; 12.7% lower, 8.2–16.9, for 9–16 weeks vs. 2–4 weeks, and 7.8% lower, 1.3–16.1, for >16 weeks vs. 2–4 weeks); shorter interval between first and second vaccine doses (10.4% lower, 3.7–16.7, for <6 weeks vs. >10 weeks; 5.9% lower, 3.3–8.3, for 6–10 weeks vs. >10 weeks); receiving the second vaccine dose during the first quarter (Q1) or the final quarter (Q4) of the year (8.1% lower, 3.2–12.7, for Q1 vs. Q2, and 47.7% lower, 11.4–69.1, for Q4 vs. Q2); older age (3.3% lower per 10-year increase in age, 2.1–4.6), and presence of hypertension (4.1% lower, 1.1–6.9). Two factors independently associated with higher post-vaccination antibody titres: South Asian ethnicity (16.2% higher, 3.0–31.1, vs. White ethnicity) or Mixed/Multiple/Other ethnicity (11.8% higher, 2.9–21.6, vs. White ethnicity), and pre-vaccination seropositivity for SARS-CoV-2 (39.8% higher, 34.7–45.0, for individuals who were seropositive without COVID-19 symptoms pre-vaccination vs. those who were seronegative pre-vaccination, and 105.1% higher, 94.1–116.6, for individuals who were seropositive with COVID-19 symptoms pre-vaccination vs. those who were seronegative pre-vaccination). 

All the above determinants of post-vaccination antibody titres remained significant when we performed a sensitivity analysis excluding 23 individuals who reported RT-PCR- or lateral flow test-confirmed SARS-CoV-2 infection after their second vaccine dose, but before their serology sampling date (data not shown).

### 3.3. Stratification of Antibody Responses by Vaccine Type

To explore whether determinants of antibody responses to vaccination were consistent for ChAdOx1 vs. BNT162b2, we stratified the analysis of post-vaccination antibody titres according to type of vaccine received. Tables S3 and S4 (Online Data Supplement) present the results of these analyses. Seven factors associated independently with post-vaccination antibody titres in ChAdOx1 recipients, but not in BNT162b2 recipients: lower titres in this group were associated with active cancer, consumption of selenium supplements and use of statins, Angiotensin Converting Enzyme (ACE) inhibitors and proton pump inhibitors (PPI), while South Asian and Mixed/Multiple/Other ethnic origin associated with higher titres. Conversely, three factors associated independently with lower post-vaccination antibody titres in BNT162b2 recipients, but not in ChAdOx1 recipients: older age, hypertension and use of systemic immunosuppressant medication. To test whether vaccine type modified the effects of these independent variables on post-vaccination antibody titres, we fitted all significant factors in our main multivariable model and included an interaction term for vaccine type. Three factors showed a small but statistically significant interaction: hypertension (P_interaction_ = 0.035), regular consumption of selenium supplements (P_interaction_ = 0.021) and use of systemic immunosuppressant medication (P_interaction_ = 0.046; Figure S2, Online Data Supplement).

### 3.4. Influence of Post-Vaccination Paracetamol/NSAIDs on Antibody Response to Primary Course of Vaccination

Results of an exploratory analysis to determine the influence of taking paracetamol or NSAIDs to treat post-vaccination symptoms are presented in Table S5. After fitting post-vaccination paracetamol/NSAID use into the main multivariable model we found a significant positive association between this factor and post-vaccination antibody titres. We then reasoned that this association could be confounded by an association between presence or severity of post-vaccination symptoms and higher post-vaccination antibody titres. After additionally correcting for report of post-vaccination symptoms (i.e., headache, fever, local soreness), the association between post-vaccination paracetamol/NSAID use and post-vaccination titres was rendered statistically non-significant.

## 4. Discussion

We present results of a large population-based study examining a comprehensive range of potential determinants of combined IgG, IgA and IgM responses to two very widely used SARS-CoV-2 vaccines. A major finding was that 5.8% of participants did not have detectable IgG/A/M anti-spike antibodies following two doses of ChAdOx1, as compared with 1.2% of those who had received two doses of BNT162b2 (aOR 6.62, 95% CI 4.21 to 10.42). Other risk factors for post-vaccination seronegativity included shorter inter-dose interval, administration during colder months, older age, poorer general health, immunodeficiency disorder and use of systemic immunosuppressive medication. By contrast, pre-vaccination SARS-CoV-2 seropositivity and regular use of vitamin D supplements at the time of vaccination were associated with lower risk of post-vaccination seronegativity. Post-vaccination antibody titres were higher in participants of South Asian and mixed/other ethnic origin vs. White participants, and in those who were overweight vs. normal weight; these associations were independent of pre-vaccination SARS-CoV-2 serostatus. Post-vaccination use of paracetamol or NSAIDs was not associated with lower antibody responses to vaccination, after adjustment for post-vaccination symptoms, and determinants of post-vaccination anti-spike titres were not substantively different for participants who received two doses of ChAdOx1 vs. BNT162b2. 

Our findings confirm previous reports of higher antibody responses to BNT162b2 vs. ChAdOx1 [4,13,26]. Pre-existing immunity to adenovirus vectors may be one of the factors underlying this difference [27], and experiments to compare the prevalence of adenovirus neutralising antibody in pre-vaccination serum of participants who did vs. did not develop anti-spike antibodies in response to ChAdOx1 are planned. 

Our results with respect to the adverse influence of older age, shorter inter-dose interval, poorer general health, immunodeficiency and immunosuppressants on post-vaccination antibody responses are also broadly consistent with those of others [4,5,6,7,8,9]–as is the finding that pre-vaccination SARS-CoV-2 infection associates with higher antibody titres after vaccination [9,12,13]. However, we also report a number of novel findings. The independent association between regular use of vitamin D supplements and reduced risk of post-vaccination seronegativity echoes results of a previous study where we showed that high-dose vitamin D replacement enhances antigen-specific cellular responses following vaccination against varicella zoster virus [28]; an intervention study nested within a randomised controlled trial of vitamin D supplementation for prevention of COVID-19 (ClinicalTrials.gov identifier NCT04579640) is currently on-going to explore this finding further. With respect to season, we found that anti-S titres were significantly higher for participants who received their second vaccine dose in Q1 vs. Q2. Seasonal variations in human immune responses are well recognised, with studies in Europeans showing a more pro-inflammatory transcriptomic profile in winter vs. summer [29,30]. Linder and colleagues reported a stronger immune response to rubella vaccine in children vaccinated in winter vs. summer in Israel [31], while Martins and colleagues found that children in Guinea-Bissau who were vaccinated against measles during the rainy season had higher antibody levels than those vaccinated in the dry season [32]. However, other investigators have found no differences in antibody responses according to season of vaccination against tetanus, diphtheria or measles or rubella [33,34]. Further research is needed to investigate the mechanisms by which season of inoculation may influence vaccine immunogenicity.

We also found that White ethnicity and lower BMI both associated with lower post-vaccination antibody titres, independently of pre-vaccination SARS-CoV-2 serostatus. Ethnic variation in SARS-CoV-2 vaccine immunogenicity has not been widely studied to date: one study has reported lower antibody responses to vaccination in Jewish vs. non-Jewish health care workers in Israel [9], while a population-based UK study reported higher antibody titres in ‘non-Whites’ vs. Whites. However, pre-vaccination serostatus was not available for all participants in these studies; this omission is potentially important, because unvaccinated people of South Asian ethnic origin were at higher risk of SARS-CoV-2 infection in the pre-vaccination era [20,24]. We demonstrate for the first time that higher anti-spike titres among vaccinated people of South Asian origin are not attributable to the higher rates of pre-vaccination SARS-CoV-2 infection that we have previously reported [20,24]. The reasons for this phenomenon require further investigation: ethnic variation in recognition of SARS-CoV-2 T cell epitopes is recognised [35], and it may be that analogous variation in B cell epitopes underlies the ethnic differences in antibody responses to vaccination seen here. By contrast with ethnicity, several studies have investigated the impact of BMI on post-vaccination antibody titres. Their results are inconsistent, with one reporting lower antibody responses in those with BMI > 30 kg/m^2^ [4], another reporting higher responses in those with higher BMI [36] and two reporting no association [37,38]. Of note, studies investigating antibody responses to influenza vaccination report higher titres initially in obese participants, but a greater decline thereafter [39]. This finding highlights the importance of longer term follow-up to elucidate the influence of BMI on humoral responses to vaccination. 

We also report some important null results. The lack of association between time of day of vaccination and degree of antibody response conflicts with findings of studies which variously report higher anti-spike responses in those vaccinated earlier [40] or later [41] in the day. We also show no association between pre- or post-vaccination use of paracetamol or NSAIDs and antibody responses, after adjustment for incidence of post-vaccination symptoms. This provides reassurance that use of these medications to manage reactive post-vaccination symptoms does not compromise SARS-CoV-2 vaccine immunogenicity. We also show no association between higher titres and socioeconomic deprivation, as reported by others [4,7]; this may reflect the fact that other studies did not adjust for pre-vaccination serostatus, which is more likely to be positive in socioeconomically deprived populations. There was no evidence of association for lifestyle factors hypothesised to influence vaccine responses including dietary factors, use of micronutrient supplements other than vitamin D, sleep, alcohol use and tobacco smoking. 

Our study has several strengths. We utilised a CE-marked semi-quantitative assay with high sensitivity and specificity that targets three different types of antibody [21], and that we have validated as a correlate of protection against breakthrough disease [22,23]. Its large size affords power to detect determinants of having undetectable anti-spike antibodies after SARS-CoV-2 vaccination–a relatively rare, but potentially clinically important outcome. The population-based nature of this study, and the fact that we investigated effects of two major vaccines utilising differing technology (one live vector, one mRNA) enhances the generalisability of our findings. Very detailed characterisation of participants allowed us to investigate a wide range of potential determinants of vaccine immunogenicity, and to adjust for multiple confounders, including pre-vaccination serostatus. 

Our study also has some limitations. We did not study cellular responses to vaccination, and these may be discordant with humoral responses [10,26,42,43,44]. Methods to detect SARS-CoV-2-specific T cell responses have been used by other groups [45,46] We also studied humoral responses at an early time point only; a high early peak in antibody titres following vaccination is not necessarily an indicator of durable response [39]. As with any observational study, we cannot exclude the possibility that some of the associations we report might be explained by residual or unmeasured confounding. However, we have minimised the risk of this by adjusting for a comprehensive list of putative determinants of vaccine immunogenicity. COVIDENCE UK is also a self-selected cohort, and thus certain demographics—such as people < 30 years old, people of lower socioeconomic status, and non-White ethnic groups—are under-represented. This may have limited our power to investigate antibody responses in some sub-groups, such as participants of Black ethnicity (who are at higher risk of SARS-CoV-2 infection [47,48,49] and adverse outcomes [50] than White people). 

In conclusion, this large population-based study reports on the influence of a comprehensive range of potential sociodemographic, behavioural, clinical, pharmacologic and nutritional determinants on antibody responses to two major SARS-CoV-2 vaccines. 

## Figures and Tables

**Table 1:** Participant characteristics, by vaccine type and overall (*n* = 9101).

Characteristic		ChAdOx1 (*n* = 5770)	BNT162b2 (*n* = 3331)	Overall (*n* = 9101)
Age	Median age, years (IQR)	63.5 (57.0–68.9)	65.5 (57.4–71.6)	64.2 (57.1–69.9)
	Age range, years	17.4 89.4	16.6 90.5	16.6 90.5
Sex, *n* (%)	Male	1671 (29.0)	956 (28.7)	2627 (28.9)
	Female	4099 (71.0)	2375 (71.3)	6474 (71.1)
Ethnicity, *n* (%) ^1^	White	5565 (96.4)	3222 (96.7)	8787 (96.5)
	South Asian	119 (2.1)	60 (1.8)	179 (2)
	Black/African/Caribbean/Black British	62 (1.1)	38 (1.1)	100 (1.1)
	Mixed/Multiple/Other	24 (0.4)	10 (0.3)	34 (0.4)
Nation of residence ^2^	England	5177 (89.8)	2906 (87.3)	8083 (88.8)
	Northern Ireland	39 (0.7)	78 (2.3)	117 (1.3)
	Scotland	358 (6.2)	199 (6.0)	557 (6.1)
	Wales	194 (3.4)	147 (4.4)	341 (3.8)
Body mass index, kg/m^2^, *n* (%) ^3^	<25	2845 (49.3)	1596 (47.9)	4441 (48.8)
	25–30	1881 (32.6)	1097 (32.9)	2978 (32.7)
	>30	1034 (17.9)	636 (19.1)	1670 (18.3)
Highest educational level attained, *n* (%) ^4^	Primary/Secondary	636 (11)	401 (12)	1037 (11.4)
	Higher/Further (A levels)	857 (14.9)	441 (13.2)	1298 (14.3)
	College	2540 (44)	1500 (45)	4040 (44.4)
	Post-graduate	1736 (30.1)	984 (29.5)	2720 (29.9)
Quantiles of IMD rank, *n* (%) ^5^	Q1 (most deprived)	1172 (20.3)	786 (23.6)	1958 (21.5)
	Q2	1417 (24.6)	782 (23.5)	2199 (24.2)
	Q3	1537 (26.6)	868 (26.1)	2405 (26.4)
	Q4 (least deprived)	1639 (28.4)	893 (26.8)	2532 (27.8)
Tobacco smoking, *n* (%)	Non-current/never smoker	5546 (96.1)	3194 (95.9)	8740 (96)
	Current smoker	224 (3.9)	137 (4.1)	361 (4)
Alcohol consumption/week, units, *n* (%)	None	1505 (26.1)	896 (26.9)	2401 (26.4)
	1–7	2018 (35)	1212 (36.4)	3230 (35.5)
	8–14	1186 (20.6)	695 (20.9)	1881 (20.7)
	15–21	593 (10.3)	310 (9.3)	903 (9.9)
	22–28	263 (4.6)	127 (3.8)	390 (4.3)
	>28	205 (3.6)	91 (2.7)	296 (3.3)
Self-assessed general health	Excellent	1183 (20.5)	654 (19.6)	1837 (20.2)
	Very good	2336 (40.5)	1315 (39.5)	3651 (40.1)
	Good	1441 (25.0)	925 (27.8)	2366 (26.0)
	Fair	637 (11.0)	344 (10.3)	981 (10.8)
	Poor	173 (3.0)	93 (2.8)	266 (2.9)
Pre-vaccination anti-spike IgG/A/M serostatus	Negative	3789 (65.7)	1770 (53.1)	5559 (61.1)
	Positive	696 (12.1)	325 (9.8)	1021 (11.2)
	Unknown	1285 (22.3)	1236 (37.1)	2521 (27.7)
Post-vaccination anti-spike IgG/A/M serostatus	Negative	334 (5.8)	40 (1.2)	374 (4.1)
	Positive	5436 (94.2)	3291 (98.8)	8727 (95.9)
Median inter-dose interval, weeks (IQR)		11.0 (10.0–11.2)	10.7 (9.5–11.1)	11.0 (9.8–11.1)
Median time from date of second vaccine dose to date of sampling, weeks (IQR)		7.6 (5.7–9.6)	10.1 (8.3–13.1)	8.6 (6.4–10.7)

Abbreviations: IQR, inter-quartile range; s.d., standard deviation; IMD, index of multiple deprivation; Ig, Immunoglobulin. (^1^) Ethnicity not reported for *n* = 1 who received BNT162b2 vaccine. (^2^) Nation of residence not reported for *n* = 1 who received BNT162b2 vaccine and *n* = 2 who received ChAdOx1 vaccine. (^3^) BMI not reported for *n* = 10 who received BNT162b2 vaccine and *n* = 2 who received ChAdOx1 vaccine. (^4^) Level of education not reported for *n* = 5 who received BNT162b2 vaccine and *n* = 1 who received ChAdOx1 vaccine. (^5^) IMD rank not reported for *n* = 2 who received BNT162b2 vaccine and *n* = 5 who received ChAdOx1 vaccine.

**Table 2:** Determinants of post-vaccination seronegativity, all participants (*n* = 9101).

Predictor		*n* Seronegative (%)	Minimally Adjusted Odds Ratio (95% CI) ^1^	Pairwise *p* Value	Fully Adjusted Odds Ratio (95% CI) ^2^	Pairwise *p* Value	P for Trend
Vaccine type and timing							
Vaccine type	ChAdOx1	334/5770 (5.8)	5.49 (3.94, 7.66)	<0.001	6.62 (4.21, 10.42)	<0.001 *	-
	BNT162b2	40/3331 (1.2)	Referent				
Time from date of second vaccine dose to date of sampling, weeks (IQR)	<2	3/34 (8.8)	2.85 (0.80, 10.20)	0.11			
	2–4	19/593 (3.2)	Referent				
	5–8	127/3878 (3.3)	0.87 (0.53, 1.42)	0.57			
	9–16	213/4155 (5.1)	1.27 (0.78, 2.07)	0.33			
	>16	12/441 (2.7)	0.68 (0.33, 1.43)	0.31			
Inter-dose interval, weeks	<6	25/474 (5.3)	1.52 (0.99, 2.34)	0.054	2.56 (1.22, 5.37)	0.013 *	<0.001
	6–10	142/2959 (4.8)	1.47 (1.17, 1.83)	0.001	1.60 (1.20, 2.14)	0.001 *	
	>10	207/5668 (3.7)	Referent				
Time of second vaccine dose	Before 12 p.m.	148/3692 (4.0)	Referent				
	12 p.m.–2 p.m.	74/1561 (4.7)	1.21 (0.91, 1.61)	0.19			
	2 p.m.–5 p.m.	103/2573 (4.0)	1.01 (0.78, 1.31)	0.92			
	After 5 p.m.	30/941 (3.2)	0.84 (0.57, 1.26)	0.41			
Quarter of second vaccine dose	Q1	56/1318 (4.2)	1.02 (0.76, 1.37)	0.87	1.08 (0.61, 1.92)	0.79	0.005 †
	Q2	313/7755 (4.0)	Referent				
	Q3	2/13 (15.4)	4.10 (0.89, 18.77)	0.07	12.64 (2.21, 72.27)	0.004 *	
	Q4	3/15 (20.0)	5.82 (1.60, 21.17)	0.007	21.83 (1.74, 273.33)	0.017 *	
Socio-demographic factors							
Age, years	<30	3/83 (3.6)	Referent				<0.001
	30–39.99	6/192 (3.1)	0.87 (0.21, 3.57)	0.85	0.96 (0.09, 10.65)	0.97	
	40–49.99	11/595 (1.8)	0.51 (0.14, 1.85)	0.30	0.53 (0.06, 4.97)	0.58	
	50–59.99	62/2266 (2.7)	0.75 (0.23, 2.44)	0.63	0.88 (0.10, 7.41)	0.90	
	60–69.99	152/3706 (4.1)	1.11 (0.35, 3.55)	0.86	1.64 (0.20, 13.67)	0.65	
	70–79.99	131/2077 (6.3)	1.69 (0.52, 5.42)	0.38	2.86 (0.34, 24.18)	0.33	
	≥80	9/182 (4.9)	1.25 (0.33, 4.76)	0.74	3.38 (0.25, 46.30)	0.36	
Sex	Female	234/6474 (3.6)	Referent				
	Male	140/2627 (5.3)	1.33 (1.07, 1.66)	0.01	1.32 (0.98, 1.77)	0.06	-
Ethnicity	White	368/8787 (4.2)	Referent				
	Mixed/Multiple/Other	4/179 (2.2)	0.59 (0.22, 1.59)	0.30			
	South Asian	1/100 (1.0)	0.27 (0.04, 1.94)	0.19			
	Black/African/Caribbean/ Black British	1/34 (2.9)	0.85 (0.12, 6.28)	0.88			
BMI, kg/m^2^	<25	164/4441 (3.7)	Referent				0.71
	25–30	135/2978 (4.5)	1.20 (0.95, 1.52)	0.13	1.15 (0.85, 1.55)	0.37	
	>30	74/1670 (4.4)	1.30 (0.98, 1.72)	0.07	1.05 (0.70, 1.56)	0.82	
Highest educational level attained	Primary/Secondary	54/1036 (5.2)	1.12 (0.80, 1.57)	0.50			
	Higher/further (A levels)	56/1298 (4.3)	0.99 (0.72, 1.38)	0.97			
	College	150/4041 (3.7)	0.86 (0.67, 1.10)	0.23			
	Post-graduate	114/2720 (4.2)	Referent				
Quantiles of IMD rank	Q1 (most deprived)	85/1958 (4.3)	1.20 (0.89, 1.61)	0.24			
	Q2	94/2199 (4.3)	1.12 (0.84, 1.49)	0.45			
	Q3	96/2405 (4.0)	1.01 (0.76, 1.35)	0.92			
	Q4 (least deprived)	99/2532 (3.9)	Referent				
Lifestyle factors							
Tobacco smoking	No	363/8740 (4.2)	Referent				
	Yes	11/361 (3.0)	0.77 (0.42, 1.42)	0.40			
Vaping	No	364/8881 (4.1)	Referent				
	Yes	9/196 (4.6)	1.16 (0.59, 2.29)	0.67			
Alcohol, units/week	None	118/2401 (4.9)	Referent				0.81
	1–7	103/3230 (3.2)	0.63 (0.48, 0.82)	0.001	0.71 (0.50, 1.01)	0.06	
	8–14	84/1880 (4.5)	0.85 (0.64, 1.14)	0.29	1.10 (0.76, 1.60)	0.61	
	15–21	40/903 (4.4)	0.83 (0.57, 1.20)	0.31	1.00 (0.63, 1.60)	0.99	
	22–28	17/391 (4.3)	0.78 (0.46, 1.32)	0.35	1.15 (0.61, 2.17)	0.68	
	>28	12/296 (4.1)	0.71 (0.38, 1.30)	0.27	0.60 (0.25, 1.46)	0.26	
Light exercise, hours/week	0–4	140/2824 (5.0)	1.52 (1.18, 1.96)	0.001	1.19 (0.86, 1.67)	0.30	0.31
	5–9	120/2987 (4.0)	1.20 (0.93, 1.56)	0.17	1.09 (0.79, 1.50)	0.61	
	≥10	114/3268 (3.5)	Referent				
Vigorous exercise, hours/week	0	155/3458 (4.5)	1.19 (0.91, 1.56)	0.20			
	1–3	132/3387 (3.9)	1.04 (0.79, 1.37)	0.79			
	≥4	87/2230 (3.9)	Referent				
Sleep, hours/night	≤5	43/797 (5.4)	1.26 (0.87, 1.81)	0.22	1.29 (0.81, 2.05)	0.29	0.54
	6	93/2215 (4.2)	0.93 (0.70, 1.23)	0.60	0.91 (0.63, 1.32)	0.62	
	7	133/3750 (3.6)	0.77 (0.59, 1.00)	0.05	0.84 (0.60, 1.17)	0.29	
	≥8	105/2336 (4.5)	Referent				
Self-assessed general health	Excellent	52/1837 (2.8)	Referent				0.002
	Very good	136/3652 (3.7)	1.31 (0.95, 1.82)	0.10	1.57 (1.03, 2.40)	0.036	
	Good	100/2365 (4.2)	1.54 (1.09, 2.16)	0.01	1.87 (1.19, 2.95)	0.007 *	
	Fair	60/981 (6.1)	2.35 (1.60, 3.44)	<0.001	2.00 (1.15, 3.47)	0.014 *	
	Poor	26/266 (9.8)	3.85 (2.35, 6.28)	<0.001	3.12 (1.39, 6.96)	0.006 *	
Anxiety or depression	No	288/6910 (4.2)	Referent				
	Yes	86/2187 (3.9)	1.00 (0.78, 1.29)	0.98			
Food choice	None	362/8607 (4.2)	Referent				
	Vegetarian	9/380 (2.4)	0.59 (0.30, 1.15)	0.12			
	Vegan	3/114 (2.6)	0.63 (0.20, 2.01)	0.44			
Medical conditions							
Heart disease ^3^	No	351/8707 (4.0)	Referent				
	Yes	23/394 (5.8)	1.26 (0.81, 1.97)	0.30			
Arterial disease ^4^	No	340/8572 (4.0)	Referent				
	Yes	34/529 (6.4)	1.45 (1.00, 2.11)	0.05	0.50 (0.23, 1.09)	0.08	-
Hypertension	No	246/6902 (3.6)	Referent				
	Yes	128/2199 (5.8)	1.56 (1.25, 1.95)	<0.001	0.89 (0.58, 1.37)	0.61	-
Immunodeficiency disorder ^5^	No	364/9042 (4.0)	Referent				
	Yes	10/59 (16.9)	4.62 (2.32, 9.23)	<0.001	6.48 (2.53, 16.59)	<0.001 *	-
Major neurological condition ^6^	No	350/8833 (4.0)	Referent				
	Yes	24/268 (9.0)	2.23 (1.44, 3.44)	<0.001	1.79 (0.82, 3.91)	0.15	-
Cancer	Never	330/8128 (4.1)	Referent				
	Past (cured or in remission)	40/888 (4.5)	1.04 (0.74, 1.47)	0.80			
	Present (active)	4/85 (4.7)	1.00 (0.36, 2.77)	0.99			
Asthma	No	303/7652 (4.0)	Referent				
	Yes	71/1449 (4.9)	1.30 (1.00, 1.70)	0.05	0.99 (0.69, 1.41)	0.94	-
COPD	No	364/8905 (4.1)	Referent				
	Yes	10/196 (5.1)	1.19 (0.62, 2.27)	0.60			
Diabetic status	No diabetes	317/8334 (3.8)	Referent				0.06
	Pre-diabetes	16/296 (5.4)	1.38 (0.82, 2.31)	0.23	1.12 (0.57, 2.22)	0.74	
	Type 1 diabetes	4/69 (5.8)	1.59 (0.57, 4.41)	0.37	1.87 (0.52, 6.75)	0.34	
	Type 2 diabetes	34/385 (8.8)	2.26 (1.56, 3.28)	<0.001	2.02 (0.94, 4.33)	0.07	
Atopy ^7^	No	281/6794 (4.1)	Referent				
	Yes	93/2307 (4.0)	1.01 (0.80, 1.29)	0.92			
Pre-vaccination SARS-CoV-2 status	Seronegative	226/5422 (4.2)	Referent				<0.001
	Seropositive, asymptomatic	18/722 (2.5)	0.58 (0.35, 0.94)	0.03	0.53 (0.32, 0.89)	0.015 *	
	Seropositive, symptomatic	2/264 (0.8)	0.20 (0.05, 0.81)	0.02	0.16 (0.04, 0.56)	0.004 *	
Nutritional supplements							
Multivitamin	No	299/7200 (4.2)	Referent				
	Yes	75/1901 (3.9)	0.98 (0.75, 1.26)	0.86			
Vitamin A	No	371/9053 (4.1)	Referent				
	Yes	3/48 (6.3)	1.56 (0.48, 5.04)	0.46			
Vitamin C	No	346/8192 (4.2)	Referent				
	Yes	28/909 (3.1)	0.73 (0.49, 1.08)	0.11			
Vitamin D	No	204/4455 (4.6)	Referent				
	Yes	170/4646 (3.7)	0.80 (0.65, 0.98)	0.03	0.66 (0.51, 0.87)	0.003 *	-
Zinc	No	357/8664 (4.1)	Referent				
	Yes	17/437 (3.9)	0.94 (0.57, 1.55)	0.82			
Selenium	No	372/9003 (4.1)	Referent				
	Yes	2/98 (2.0)	0.49 (0.12, 2.01)	0.32			
Iron	No	368/8816 (4.2)	Referent				
	Yes	6/285 (2.1)	0.53 (0.23, 1.19)	0.12			
Probiotics	No	359/8528 (4.2)	Referent				
	Yes	15/573 (2.6)	0.64 (0.38, 1.09)	0.10			
Omega-3 fatty acids	No	336/7975 (4.2)	Referent				
	Yes	38/1126 (3.4)	0.80 (0.57, 1.12)	0.19			
Cod liver oil	No	341/8279 (4.1)	Referent				
	Yes	33/822 (4.0)	0.93 (0.64, 1.34)	0.68			
Garlic	No	364/8907 (4.1)	Referent				
	Yes	10/194 (5.2)	1.20 (0.63, 2.30)	0.57			
Medications							
Beta-2 adrenergic agonists	No	334/8275 (4.0)	Referent				
	Yes	40/826 (4.8)	1.23 (0.87, 1.72)	0.24			
Beta blockers	No	334/8398 (4.0)	Referent				
	Yes	40/703 (5.7)	1.35 (0.96, 1.89)	0.09	1.01 (0.62, 1.65)	0.96	-
Statins	No	277/7337 (3.8)	Referent				
	Yes	97/1764 (5.5)	1.31 (1.02, 1.68)	0.03	0.92 (0.64, 1.32)	0.65	-
ACE inhibitors	No	320/8122 (3.9)	Referent				
	Yes	54/979 (5.5)	1.30 (0.96, 1.76)	0.09	1.12 (0.70, 1.80)	0.64	-
Proton pump inhibitors	No	287/7723 (3.7)	Referent				
	Yes	87/1378 (6.3)	1.69 (1.32, 2.16)	<0.001	0.91 (0.63, 1.33)	0.63	-
H2-receptor antagonists	No	373/9040 (4.1)	Referent				
	Yes	1/61 (1.6)	0.39 (0.05, 2.80)	0.35			
Inhaled corticosteroids	No	349/8505 (4.1)	Referent				
	Yes	25/596 (4.2)	1.01 (0.66, 1.53)	0.98			
Bronchodilators	No	333/8240 (4.0)	Referent				
	Yes	41/861 (4.8)	1.20 (0.85, 1.67)	0.30			
Systemic Immunosuppressants	No	327/8630 (3.8)	Referent				
	Yes	47/471 (10.0)	2.86 (2.08, 3.95)	<0.001	3.71 (2.41, 5.69)	<0.001 *	-
Angiotensin receptor blockers	No	331/8493 (3.9)	Referent				
	Yes	43/608 (7.1)	1.75 (1.25, 2.43)	<0.001	0.98 (0.58, 1.68)	0.95	-
SSRI antidepressants	No	350/8533 (4.1)	Referent				
	Yes	24/568 (4.2)	1.11 (0.73, 1.70)	0.62			
Non-SSRIs antidepressants	No	352/8726 (4.0)	Referent				
	Yes	22/375 (5.9)	1.57 (1.01, 2.46)	0.05	0.73 (0.35, 1.50)	0.39	-
Calcium channel blockers	No	314/8100 (3.9)	Referent				
	Yes	60/1001 (6.0)	1.45 (1.09, 1.94)	0.01	1.00 (0.65, 1.55)	0.99	-
Thiazide diuretics	No	359/8765 (4.1)	Referent				
	Yes	15/336 (4.5)	1.04 (0.61, 1.76)	0.89			
Vitamin K antagonists	No	370/9027 (4.1)	Referent				
	Yes	4/74 (5.4)	1.17 (0.42, 3.23)	0.76			
SGLT-2 inhibitors	No	369/9054 (4.1)	Referent				
	Yes	5/47 (10.6)	2.56 (1.01, 6.54)	0.05	2.99 (0.92, 9.65)	0.07	-
Anticholinergics	No	354/8656 (4.1)	Referent				
	Yes	20/445 (4.5)	1.08 (0.67, 1.72)	0.76			
Metformin	No	353/8827 (4.0)	Referent				
	Yes	21/274 (7.7)	1.83 (1.16, 2.90)	0.01	0.61 (0.24, 1.55)	0.30	-
Bisphosphonates	No	362/8912 (4.1)	Referent				
	Yes	12/189 (6.3)	1.72 (0.94, 3.12)	0.08	0.77 (0.31, 1.89)	0.57	-
Anti-platelet drugs	No	328/8453 (3.9)	Referent				
	Yes	46/648 (7.1)	1.67 (1.21, 2.32)	0.002	2.79 (1.06, 7.38)	0.038	-
Sex hormone therapy	No	347/8376 (4.1)	Referent				
	Yes	27/725 (3.7)	1.04 (0.69, 1.56)	0.87			
Aspirin ^8^	No	341/8593 (4.0)	Referent				
	Yes	33/508 (6.5)	1.47 (1.01, 2.14)	0.04	0.37 (0.14, 1.01)	0.05	-
Paracetamol ^8^	No	345/8677 (4.0)	Referent				
	Yes	29/424 (6.8)	1.77 (1.19, 2.62)	0.005	0.84 (0.47, 1.51)	0.56	-
BCG vaccinated	No	44/1075 (4.1)	Referent				
	Yes	280/7166 (3.9)	0.99 (0.71, 1.37)	0.13			

Abbreviations: CI, confidence interval; BMI, body mass index; IMD, index of multiple deprivation; COPD, chronic obstructive pulmonary disease; SARS-CoV-2, severe acute respiratory syndrome coronavirus 2; COVID-19, coronavirus disease 2019; ACE, angiotensin-converting enzyme; H2, histamine 2; SSRI, selective serotonin reuptake inhibitor; BCG, Bacille Calmette-Guérin; SGLT2, sodium-glucose cotransporter-2. (^1^) Adjusted for age and sex only. (^2^) Adjusted for age, sex, vaccine type, inter-dose interval, quarter of second vaccination, BMI, alcohol intake, light exercise, sleep, self-assessed general health, arterial disease, hypertension, immunodeficiency disorder, major neurological condition, asthma, diabetic status, pre-vaccination SARS-CoV-2 status and pre-vaccination use of vitamin D supplements, beta blockers, statins, ACE inhibitors, proton pump inhibitors, systemic immunosuppressants, angiotensin receptor blockers, non-SSRI antidepressants, calcium channel blockers, SGLT2 inhibitors, metformin, bisphosphonates, anti-platelet drugs use, aspirin and paracetamol. (^3^) Heart disease defined as coronary artery disease or heart failure. (^4^) Arterial disease defined as ischaemic heart disease, peripheral vascular disease or cerebrovascular disease. (^5^) Immunodeficiency defined as HIV, primary immune deficiency or other immunodeficiency. (^6^) Major neurological conditions defined as stroke, transient ischaemic attack, dementia, Parkinson’s disease, multiple sclerosis or motor neurone disease. (^7^) Atopy defined as atopic eczema/dermatitis and/or hay fever/allergic rhinitis. (^8^) Chronic use prior to vaccination (i.e., distinct from acute post-vaccination use for treatment of reactogenic symptoms). † Global *p* value presented for this non-scalar categorical variable. * Below multiple comparisons testing critical *p*-value threshold of 0.024.

**Table 3:** Determinants of post-vaccination antibody titres, subset of seropositive participants (*n* = 8727).

Predictor		Median IgGAM Ratio (IQR)	Minimally Adjusted % Difference (95% CI) ^1^	*p* Value	Fully Adjusted % Difference (95% CI) ^2^	Pairwise *p* Value	P for Trend
Vaccine type	ChAdOx1	2.39 (1.76, 3.30)	−39.35 (−40.65, −38.02)	<0.001	−43.31 (−44.8, −41.78)	<0.001 *	-
	BNT162b2	3.96 (3.15, 4.86)	Referent				
Time from second vaccine dose to sampling, weeks	<2	2.96 (2.23, 13.62)	36.68 (11.82, 67.05)	0.002	1.98 (−17.97, 26.79)	0.86	<0.001
	2–4	2.86 (2.06, 4.05)	Referent				
	5–8	2.81 (1.95, 3.99)	−0.59 (−5.37, 4.43)	0.81	−7.41 (−11.62, −3.00)	0.001 *	
	9–16	3.13 (2.10, 4.24)	7.54 (2.33, 13.01)	0.004	−12.68 (−16.94, −8.20)	<0.001 *	
	>16	2.93 (2.03, 4.43)	7.14 (−0.08, 14.89)	0.05	−7.83 (−16.12, 1.28)	0.09	
Inter−dose interval, weeks	<6	2.91 (1.94, 4.12)	−1.23 (−6.38, 4.21)	0.65	−10.43 (−16.69, −3.70)	0.003 *	<0.001
	6–10	2.89 (2.00, 4.01)	−5.12 (−7.51, −2.66)	<0.001	−5.85 (−8.3, −3.34)	<0.001 *	
	>10	2.99 (2.06, 4.21)	Referent				
Time of second vaccine dose	Before 12 p.m.	2.92 (2.02, 4.10)	Referent				0.96 †
	12 p.m.–2 p.m.	2.91 (2.00, 4.07)	−1.12 (−4.39, 2.26)	0.51	−2.87 (−6.00, 0.36)	0.08	
	2 p.m.–5 p.m.	3.03 (2.06, 4.21)	2.88 (−0.01, 5.86)	0.05	1.15 (−1.61, 3.99)	0.42	
	After 5 p.m.	2.98 (2.07, 4.07)	0.54 (−3.45, 4.70)	0.79	−2.39 (−6.2, 1.58)	0.24	
Quarter of second vaccine dose	Q1	3.44 (2.42, 4.43)	15.46 (11.70, 19.34)	<0.001	−8.07 (−12.69, −3.22)	0.001 *	0.004 †
	Q2	2.88 (1.99, 4.09)	Referent				
	Q3	3.17 (1.36, 4.47)	−3.37 (−30.38, 34.12)	0.84	31.63 (−22.10, 122.40)	0.30	
	Q4	1.93 (1.28, 3.24)	−24.08 (−44.54, 3.92)	0.09	−47.69 (−69.11, −11.39)	0.016 *	
Age, years	<30	3.79 (2.48, 4.85)	Referent				<0.001
	30–39.99	3.43 (2.34, 4.55)	−12.71 (−24.56, 1.00)	0.07	−5.94 (−20.45, 11.21)	0.47	
	40–49.99	3.13 (2.16, 4.31)	−16.59 (−26.77, −5.01)	0.006	−4.12 (−17.60, 11.58)	0.59	
	50–59.99	3.10 (2.14, 4.30)	−16.58 (−26.32, −5.55)	0.004	0.70 (−13.04, 16.61)	0.93	
	60–69.99	2.85 (1.98, 4.05)	−22.79 (−31.75, −12.65)	<0.001	−5.39 (−18.27, 9.53)	0.46	
	70–79.99	2.92 (1.96, 4.03)	−23.08 (−32.09, −12.87)	<0.001	−10.54 (−22.81, 3.69)	0.14	
	≥80	2.80 (1.84, 3.78)	−24.71 (−35.06, −12.72)	<0.001	−19.62 (−34.77, −0.95)	0.040	
Sex	Female	3.01 (2.07, 4.19)	Referent				
	Male	2.87 (1.95, 4.04)	−3.17 (−5.68, −0.58)	0.02	−2.48 (−5.05, 0.17)	0.07	-
Ethnicity	White	2.94 (2.02, 4.11)	Referent				<0.001 †
	Mixed/Multiple/Other	3.35 (2.24, 4.73)	12.78 (3.75, 22.59)	0.005	11.83 (2.85, 21.59)	0.009 *	
	South Asian	3.46 (2.58, 4.64)	16.26 (4.10, 29.85)	0.008	16.21 (3.02, 31.10)	0.015 *	
	Black/African/ Caribbean/Black British	3.54 (2.39, 5.46)	26.76 (4.79, 53.33)	0.015	12.31 (−6.94, 35.54)	0.23	
BMI, kg/m^2^	<25	2.90 (2.03, 4.03)	Referent				0.038
	25–30	2.96 (2.04, 4.15)	3.62 (0.91, 6.40)	0.009	2.89 (0.18, 5.67)	0.037	
	>30	3.17 (2.02, 4.36)	4.87 (1.56, 8.29)	0.004	2.62 (−0.85, 6.21)	0.14	
Highest educational level attained	Primary/Secondary	3.02 (1.93, 4.29)	1.05 (1.01, 1.10)	0.018	1.73 (−2.36, 5.99)	0.41	0.37
	Higher/further (A levels)	2.98 (2.07, 4.14)	1.03 (1.00, 1.07)	0.07	1.59 (−2.14, 5.45)	0.41	
	College	2.99 (2.07, 4.11)	1.04 (1.01, 1.07)	0.009	3.31 (0.52, 6.17)	0.020 *	
	Post-graduate	2.89 (1.98, 4.10)	Referent				
Quantiles of IMD rank	Q1 (most deprived)	3.06 (2.12, 4.24)	2.28 (−1.10, 5.79)	0.19			
	Q2	2.97 (1.98, 4.19)	0.53 (−2.68, 3.85)	0.75			
	Q3	2.90 (2.04, 4.01)	−1.34 (−4.42, 1.84)	0.41			
	Q4 (least deprived)	2.95 (2.01, 4.14)	Referent				
Tobacco smoking	No	2.95 (2.03, 4.13)	Referent				
	Yes	3.20 (2.24, 4.20)	1.22 (−4.65, 7.46)	0.69			
Vaping	No	2.95 (2.03, 4.13)	Referent				
	Yes	3.37 (2.18, 4.37)	5.47 (−2.73, 14.37)	0.20			
Alcohol, units/week	None	2.97 (2.05, 4.20)	Referent				0.71
	1–7	3.03 (2.06, 4.14)	−0.68 (−3.63, 2.35)	0.66	1.36 (−1.66, 4.48)	0.38	
	8–14	2.94 (2.02, 4.16)	−1.87 (−5.21, 1.58)	0.28	0.9 (−2.57, 4.48)	0.62	
	15–21	2.91 (1.98, 4.01)	−3.31 (−7.46, 1.03)	0.13	0.3 (−3.97, 4.76)	0.89	
	22–28	2.65 (1.91, 3.94)	−6.32 (−11.90, −0.39)	0.04	−4.21 (−9.79, 1.71)	0.16	
	>28	2.84 (2.03, 4.18)	−3.06 (−9.56, 3.90)	0.38	0.01 (−6.59, 7.07)	1.00	
Light exercise, hours/week	0–4	3.07 (2.10, 4.26)	5.22 (2.21, 8.31)	0.001	0.18 (−2.81, 3.26)	0.91	0.92
	5–9	2.95 (2.02, 4.13)	1.29 (−1.54, 4.20)	0.38	−1.02 (−3.77, 1.82)	0.48	
	≥10	2.88 (1.99, 4.06)	Referent				
Vigorous exercise, hours/week	0	3.02 (2.05, 4.21)	4.97 (1.82, 8.21)	0.002	1.34 (−1.82, 4.6)	0.41	0.45
	1–3	2.98 (2.05, 4.12)	3.09 (−0.01, 6.28)	0.05	0.01 (−2.96, 3.06)	1.00	
	≥4	2.86 (1.99, 4.06)	Referent				
Sleep, hours/night	≤5	3.07 (2.13, 4.29)	5.03 (0.31, 9.98)	0.04	0.64 (−3.96, 5.45)	0.79	0.94
	6	3.00 (2.04, 4.19)	1.12 (−2.18, 4.52)	0.51	−1.20 (−4.41, 2.11)	0.47	
	7	2.92 (2.00, 4.08)	−1.82 (−4.66, 1.11)	0.22	−2.48 (−5.28, 0.39)	0.39	
	≥8	2.96 (2.05, 4.13)	Referent				
Self−assessed general health	Excellent	2.92 (2.05, 4.07)	Referent				0.27
	Very good	2.92 (2.01, 4.09)	−0.20 (−3.32, 3.02)	0.90	−2.35 (−5.36, 0.75)	0.14	
	Good	3.03 (2.06, 4.23)	4.73 (1.17, 8.42)	0.009	−0.86 (−4.34, 2.74)	0.63	
	Fair	3.07 (2.02, 4.15)	2.38 (−2.07, 7.03)	0.30	−1.96 (−6.45, 2.74)	0.41	
	Poor	2.95 (2.04, 4.39)	5.60 (−2.04, 13.84)	0.16	−6.73 (−14.02, 1.17)	0.09	
Anxiety or depression	No	2.95 (2.03, 4.13)	Referent				
	Yes	2.99 (2.03, 4.17)	0.68 (−2.06, 3.50)	0.63			
Food choice	None	2.96 (2.03, 4.13)	Referent				
	Vegetarian	2.92 (2.04, 4.13)	−2.98 (−8.46, 2.83)	0.31			
	Vegan	2.61 (2.00, 4.33)	−2.04 (−11.76, 8.74)	0.70			
Heart disease ^3^	No	2.97 (2.03, 4.13)	Referent				
	Yes	2.76 (1.93, 4.17)	1.33 (−4.46, 7.48)	0.66			
Arterial disease ^4^	No	2.96 (2.04, 4.13)	Referent				
	Yes	2.96 (1.94, 4.14)	2.02 (−3.09, 7.40)	0.45			
Hypertension	No	2.98 (2.06, 4.16)	Referent				
	Yes	2.89 (1.92, 4.08)	−2.62 (−5.32, 0.14)	0.06	−4.09 (−6.94, −1.14)	0.007 *	-
Immunodeficiency disorder ^5^	No	2.96 (2.03, 4.13)	Referent				
	Yes	2.85 (1.98, 3.96)	−0.17 (−14.65, 16.77)	0.98			
Major neurological condition ^6^	No	2.96 (2.04, 4.13)	Referent				
	Yes	2.90 (1.84, 4.10)	−0.74 (−7.55, 6.57)	0.84			
Cancer	Never	2.95 (2.04, 4.13)	Referent				
	Past (cured or in remission)	2.99 (2.00, 4.15)	1.24 (−2.72, 5.35)	0.55			
	Present (active)	2.93 (1.89, 3.82)	−5.33 (−16.23, 7.00)	0.38			
Asthma	No	2.97 (2.03, 4.14)	Referent				
	Yes	2.91 (2.06, 4.10)	−0.74 (−3.88, 2.51)	0.65			
COPD	No	2.96 (2.03, 4.13)	Referent				
	Yes	3.06 (1.98, 4.12)	2.97 (−5.06, 11.67)	0.48			
Diabetic status	No diabetes	2.97 (2.04, 4.14)	Referent				
	Pre-diabetes	2.68 (1.87, 3.93)	−4.13 (−10.31, 2.46)	0.21			
	Type 1 diabetes	3.13 (1.95, 4.10)	−1.81 (−14.31, 12.52)	0.79			
	Type 2 diabetes	2.95 (1.88, 4.30)	0.77 (−5.09, 6.98)	0.80			
Atopy ^7^	No	2.95 (2.02, 4.14)	Referent				
	Yes	2.97 (2.05, 4.11)	−0.14 (−2.80, 2.59)	0.92			
Pre−vaccination SARS−COV−2 status	Seronegative	2.74 (1.92, 3.80)	Referent				<0.001
	Seropositive, asymptomatic	3.62 (2.40, 5.01)	37.37 (31.83, 43.13)	<0.001	39.77 (34.73, 45.00)	<0.001 *	
	Seropositive, symptomatic	5.50 (4.17, 12.62	126.30 (112.07, 141.5)	<0.001	105.06 (94.13, 116.60)	<0.001 *	
Multivitamin	No	2.97 (2.03, 4.13)	Referent				
	Yes	2.91 (2.05, 4.17)	−0.64 (−3.46, 2.26)	0.66			
Vitamin A	No	2.96 (2.03, 4.13)	Referent				
	Yes	2.90 (1.94, 3.93)	−4.11 (−18.56, 12.91)	0.61			
Vitamin C	No	2.95 (2.02, 4.11)	Referent				
	Yes	3.13 (2.10, 4.26)	4.07 (0.10, 8.19)	0.04	1.47 (−2.42, 5.52)	0.46	-
Vitamin D	No	2.98 (2.05, 4.12)	Referent				
	Yes	2.94 (2.02, 4.15)	−0.87 (−3.16, 1.49)	0.47			
Zinc	No	2.96 (2.03, 4.13)	Referent				
	Yes	2.91 (2.07, 4.18)	1.48 (−3.92, 7.18)	0.60			
Selenium	No	2.96 (2.03, 4.13)	Referent				
	Yes	2.57 (1.85, 4.06)	−6.21 (−16.16, 4.92)	0.26			
Iron	No	2.96 (2.04, 4.13)	Referent				
	Yes	2.80 (1.88, 4.10)	−3.18 (−9.42, 3.49)	0.34			
Probiotics	No	2.97 (2.03, 4.13)	Referent				
	Yes	2.87 (2.08, 4.27)	0.52 (−4.18, 5.45)	0.83			
Omega−3 fatty acids	No	2.95 (2.02, 4.13)	Referent				
	Yes	3.01 (2.12, 4.21)	3.08 (−0.51, 6.80)	0.09	2.09 (−1.48, 5.79)	0.26	-
Cod liver oil	No	2.96 (2.04, 4.13)	Referent				
	Yes	2.97 (2.00, 4.12)	1.15 (−2.90, 5.38)	0.58			
Garlic	No	2.95 (2.03, 4.13)	Referent				
	Yes	3.06 (2.26, 4.42)	6.51 (−1.83, 15.55)	0.13			
Beta−2 adrenergic agonists	No	2.96 (2.03, 4.13)	Referent				
	Yes	2.94 (2.05, 4.13)	0.83 (−3.21, 5.04)	0.69			
Beta blockers	No	2.97 (2.04, 4.13)	Referent				
	Yes	2.87 (1.94, 4.11)	−1.17 (−5.47, 3.32)	0.60			
Statins	No	2.98 (2.06, 4.17)	Referent				
	Yes	2.86 (1.91, 4.01)	−2.52 (−5.51, 0.57)	0.11			
ACE inhibitors	No	2.97 (2.05, 4.14)	Referent				
	Yes	2.89 (1.85, 4.09)	−1.85 (−5.55, 2.00)	0.34			
Proton pump inhibitors	No	2.97 (2.05, 4.13)	Referent				
	Yes	2.91 (1.92, 4.16)	0.26 (−3.00, 3.63)	0.88			
H2−receptor antagonists	No	2.96 (2.03, 4.13)	Referent				
	Yes	3.02 (2.09, 4.20)	1.57 (−11.84, 17.01)	0.83			
Inhaled corticosteroids	No	2.96 (2.03, 4.13)	Referent				
	Yes	2.88 (2.03, 4.11)	−0.44 (−5.04, 4.39)	0.86			
Bronchodilators	No	2.96 (2.03, 4.13)	Referent				
	Yes	2.97 (2.08, 4.18)	2.06 (−1.95, 6.24)	0.32			
Systemic immunosuppressants	No	2.97 (2.04, 4.14)	Referent				
	Yes	2.80 (1.99, 3.91)	−4.33 (−9.40, 1.02)	0.11			
Angiotensin receptor blockers	No	2.96 (2.04, 4.14)	Referent				
	Yes	2.94 (1.93, 4.03)	−2.33 (−6.89, 2.45)	0.33			
SSRI antidepressants	No	2.95 (2.03, 4.11)	Referent				
	Yes	3.17 (2.08, 4.33)	5.15 (0.17, 10.39)	0.04	0.18 (−4.69, 5.29)	0.94	-
Non−SSRIs antidepressants	No	2.95 (2.03, 4.13)	Referent				
	Yes	2.98 (2.01, 4.18)	2.24 (−3.66, 8.51)	0.46			
Calcium channel blockers	No	2.96 (2.05, 4.14)	Referent				
	Yes	2.94 (1.92, 4.11)	−1.54 (−5.23, 2.29)	0.43			
Thiazide diuretics	No	2.97 (2.04, 4.14)	Referent				
	Yes	2.75 (1.84, 3.95)	−5.56 (−11.27, 0.52)	0.07	−2.77 (−8.88, 3.76)	0.40	-
Vitamin K antagonists	No	2.96 (2.03, 4.14)	Referent				
	Yes	2.94 (1.89, 3.81)	−5.97 (−17.55, 7.23)	0.36			
SGLT2 inhibitors	No	2.95 (2.03, 4.13)	Referent				
	Yes	3.93 (2.44, 4.86)	10.26 (−6.90, 30.57)	0.26			
Anticholinergics	No	2.96 (2.03, 4.13)	Referent				
	Yes	3.04 (2.11, 4.18)	3.08 (−2.38, 8.84)	0.27			
Metformin	No	2.95 (2.03, 4.12)	Referent				
	Yes	3.20 (2.02, 4.46)	6.77 (−0.44, 14.50)	0.07	0.36 (−6.45, 7.66)	0.92	-
Bisphosphonates	No	2.96 (2.03, 4.13)	Referent				
	Yes	2.73 (1.99, 4.16)	−1.09 (−8.99, 7.50)	0.80			
Anti−platelet drugs	No	2.97 (2.04, 4.14)	Referent				
	Yes	2.87 (1.88, 4.05)	0.49 (−4.12, 5.32)	0.84			
Sex hormone therapy	No	2.95 (2.03, 4.13)	Referent				
	Yes	3.06 (2.08, 4.18)	0.53 (−3.77, 5.03)	0.81			
Aspirin ^8^	No	2.97 (2.04, 4.13)	Referent				
	Yes	2.84 (1.89, 4.11)	0.01 (−5.09, 5.39)	1.00			
Paracetamol ^8^	No	2.95 (2.03, 4.13)	Referent				
	Yes	3.03 (1.99, 4.23)	2.81 (−2.82, 8.76)	0.34			
BCG vaccinated	No	2.86 (2.05, 4.07)	Referent				
	Yes	3.00 (2.05, 4.17)	2.77 (−0.92, 6.59)	0.65			

Abbreviations: IQR, inter-quartile range; CI, confidence interval; BMI, body mass index; Ig, immunoglobulin; IMD, index of multiple deprivation; COPD, chronic obstructive pulmonary disease; SARS-CoV-2, severe acute respiratory syndrome coronavirus 2; COVID-19, coronavirus disease 2019; ACE, angiotensin-converting enzyme; H2, histamine 2; SSRI, selective serotonin reuptake inhibitor; BCG, Bacille Calmette-Guérin; SGLT2, sodium-glucose cotransporter-2. (^1^) Adjusted for age and sex only. (^2^) Adjusted for age, sex, vaccine type, time from second dose to sampling, inter-dose interval, time of second vaccination, quarter of second vaccination, ethnicity, BMI, educational level, alcohol intake, light exercise, vigorous exercise, sleep, self-assessed general health, hypertension, pre-vaccination SARS-CoV-2 status and pre-vaccination use of vitamin C supplements, omega-3 fatty acid supplements, SSRI antidepressants, thiazide diuretics and metformin. (^3^) Heart disease defined as coronary artery disease or heart failure. (^4^) Arterial disease defined as ischaemic heart disease, peripheral vascular disease or cerebrovascular disease. (^5^) Immunodeficiency defined as HIV, primary immune deficiency or other immunodeficiency. (^6^) Major neurological conditions defined as stroke, transient ischaemic attack, dementia, Parkinson’s disease, multiple sclerosis or motor neurone disease. (^7^) Atopy defined as atopic eczema/dermatitis and/or hay fever/allergic rhinitis. (^8^) Chronic use prior to vaccination (i.e., distinct from acute post-vaccination use for treatment of reactogenic symptoms). † Global *p* value presented for this non-scalar categorical variable. * Below multiple comparisons testing critical *p*-value threshold of 0.025.

## Data Availability

De-identified participant data will be made available upon reasonable request to the corresponding author, subject to terms of Research Ethics Committee approval and Sponsor requirements.

## Supplementary Materials

The following supporting information can be downloaded at: https://www.mdpi.com/article/10.3390/vaccines10101601/s1. Table S1. Baseline questions; Table S2. Monthly follow-up questions; Table S3. Determinants of antibody titres following administration of two doses of ChAdOx1 nCoV-19 vaccine; Table S4. Determinants of antibody titres following administration of two doses of BNT162b2 vaccine; Table S5. Influence of post-vaccination use of paracetamol/NSAIDs on antibody titres; Figure S1. Study profile; Figure S2. Post-vaccination SARS-CoV-2 antibody titres by vaccine type vs. diagnosis of hypertension (A), prescribed immunosuppressant medications (B) or consumption of selenium supplements (C) [51,52].
