# Supplementary material for: Determinants of Antibody Responses to SARS-CoV-2 Vaccines: Population-Based Longitudinal Study (COVIDENCE UK)
Source: Vaccines (Basel). 2022 Sep 23;10(10):1601. doi: 10.3390/vaccines10101601 (PMC9610049; doi:10.3390/vaccines10101601)

Determinants of antibody responses to SARS-CoV-2 vaccines: population-based longitudinal study (COVIDENCE UK)

Online Data Supplement

David A. Jolliffe [1, 2], Sian E. Faustini [3], Hayley Holt [1,2,4], Natalia Perdek [2], Sheena Maltby [2], Mohammad Talaei [1], Matthew Greenig [2], Giulia Vivaldi [1,2], Florence Tydeman [1,2], Jane Symons [5], Gwyneth A. Davies [6], Ronan A. Lyons [6], Christopher J. Griffiths [1,4], Frank Kee [7], Aziz Sheikh [8], Seif O. Shaheen [1], Alex G. Richter [3] and Adrian R. Martineau [1,2,4,*]

[1]Wolfson Institute of Population Health, Barts and The London School of Medicine and Dentistry, Queen Mary University of London, London, UK
[2]Blizard Institute, Barts and The London School of Medicine and Dentistry, Queen Mary University of London, London, UK
[3]Institute of Immunology and Immunotherapy, College of Medical and Dental Sciences, University of Birmingham, Birmingham, UK
[4]Asthma UK Centre for Applied Research, Queen Mary University of London, London, UK
[5]Jane Symons Media, London, UK
[6]Population Data Science, Swansea University Medical School, Singleton Park, Swansea, UK
[7]Centre for Public Health Research (NI), Queen's University Belfast, Belfast, UK
[8]Usher Institute, University of Edinburgh, Edinburgh, UK

[*] Corresponding author: Blizard Institute, Barts and The London School of Medicine and Dentistry, Queen Mary University of London, 4 Newark St, London E1 2AT, UK. Telephone: +44 207 882 7242. Email: a.martineau@qmul.ac.uk

Contents

Supplementary Methods 3

Procedures 3

Outcomes 4

Independent variables 4

Sample size 4

Statistical analysis 5

References 6

Supplementary Tables 7

Table S1. Baseline questions 7

Table S2. Monthly follow-up questions 11

Table S3. Determinants of antibody titres following administration of two doses of ChAdOx1 nCoV-19 vaccine. 13

Table S4. Determinants of antibody titres following administration of two doses of BNT162b2 vaccine. 20

Table S5. Influence of post-vaccination use of paracetamol/NSAIDs on antibody titres 27

Supplementary Figures 28

Figure S1. Study profile 28

Figure S2. Post-vaccination SARS-CoV-2 antibody titres by vaccine type vs. diagnosis of hypertension (A), prescribed immunosuppressant medications (B) or consumption of selenium supplements (C). 29

Supplementary Methods

Procedures

Participants were sent kits containing instructions, lancets, and blood spot collection cards, to be posted back to the study team. Once returned, the samples were logged by the study team and sent in batches to the Clinical Immunology Service at the Institute of Immunology and Immunotherapy of the University of Birmingham (Birmingham, UK) for semi-quanttative determination of combined IgG, IgM and IgA anti-spike antibody titres using a commercially available ELISA that measures combined IgG, IgA, and IgM (IgGAM) responses to the SARS-CoV-2 trimeric spike glycoprotein (product code MK654; The Binding Site [TBS], Birmingham, UK). This assay has been CE-marked with 98.3% (95% Confidence Interval [CI] 96.4–99.4) specificity and 98.6% (92.6–100.0) sensitivity following reverse transcription polymerase chain reaction (RT-PCR)-confirmed mild-to-moderate COVID-19 that did not result in hospitalisation (1). A cut-off ratio relative to the TBS cut-off calibrators was determined by plotting 624 pre-2019 negatives in a frequency histogram. A cut-off coefficient was then established for IgGAM (1.31), with ratio values classed as positive (≥1) or negative (<1). Dried blood spot eluates were pre-diluted 1:40 with 0.05% PBS-Tween using a Dynex Revelation automated absorbance microplate reader (Dynex Technologies; Chantilly, VA, USA). Plates were developed after 10 min using 3,3′,5,5′-tetramethylbenzidine core, and orthophosphoric acid used as a stop solution (both TBS). Optical densities at 450 nm were measured using the Dynex Revelation.

Outcomes

Study outcomes were presence *vs* absence of antibodies against SARS-CoV-2 (analysed as a binary outcome) and antibody titres (continuous outcome restricted to the individuals who were seropositive after receiving two vaccine doses).

Independent variables

Sixty-six putative determinants of post-vaccination serological responses were selected *a priori*, covering vaccination type and timing, sociodemographic, occupational, and lifestyle factors; longstanding medical conditions and prescribed medication use; Bacille Calmette Guérin (BCG) vaccine status, and diet and supplemental micronutrient intake (Tables S1-2, Online Data Supplement). These factors, which were obtained from the baseline or monthly questionnaires, were included as independent variables in our models. To produce participant-level covariates for each class of medications investigated, questionnaire responses were mapped to drug classes listed in the British National Formulary (BNF) or the DrugBank and Electronic Medicines Compendium databases if not explicitly listed in the BNF, as previously described (2). Index of Multiple Deprivation (IMD) 2019 scores were assigned according to participants' postcodes, and categorised into quartiles.

Sample size

Using the Stata powerlog program, we estimated that a minimum sample size of 8,192 would be required to detect a difference of at least 2% in the proportion of exposed *vs* unexposed participants experiencing a given binary outcome (equivalent to an adds

ratio [OR] of 1.08), with 80% power, for a binary exposure with maximum variability (probability 0.50 changing to 0.52) and a moderate correlation (R^2=0.4) with other variables in a logistic regression model, using a two-sided test and 5% significance. The post-vaccination antibody study was a pragmatic study including all participants meeting the inclusion criteria, with no sample size specified.

Statistical analysis

Statistical analyses were performed using Stata (version 14.2). Logistic regression models were used to estimate adjusted ORs, 95% CIs and associated pairwise P-values for potential determinants of post-vaccination seronegativity in all participants. Linear regression models were used to estimate beta-coefficients, 95% CIs and associated pairwise P-values for potential determinants of log-transformed antibody titres in the subset of seropositive participants. For ease of interpretation, log-transformed estimates of antibody titres were exponentiated and a percentage increase or decrease was calculated for every one-unit increase in the potential determinant. We first estimated ORs and beta-coefficients in minimally adjusted models, and included factors independently associated with each outcome at the 10% significance level in fully adjusted models. Both the minimally adjusted and fully adjusted models were controlled for age (<30 years, 30 to <40 years, 40 to <50 years, 50 to <60 years, 60 to <70 years, 70 to <80 years and ≥80 years) and sex (male *vs* female). For fully adjusted models, we additionally calculated a P-value for trend for scalar independent variables with more than 2 levels, or a global P-value for non-scalar independent variables with more than 2 levels. Pairwise associations having P-values <0.05 were not considered significant in fully adjusted models where the global P-value or P-value for trend for the independent variable in question was ≥0.05. Paired

comparisons of antibody titres were conducted using Wilcoxon matched pairs signed rank tests and unpaired comparisons were conducted using Mann-Whitney U tests (for 2 groups) and Kruskal Wallis tests (for three or more groups). Analyses were done for all participants with available data only; missing data were not imputed. Correction for multiple comparisons was performed on multivariable model pairwise p-values, using the Benjamini & Hochberg method with a false discovery rate (FDR) of 10% (3)

In a sensitivity analysis, we excluded participants from the analysis of antibody titres after two vaccine doses who reported a positive RT-PCR or lateral flow test for SARS-CoV-2 between the date of their second dose of vaccine and the date on which they provided their dried blood spot sample. We also stratified the analysis of antibody titres following two vaccine doses according to the type of vaccine received to explore whether determinants of antibody responses to two vaccine doses were consistent for ChAdOx1 *vs* BNT162b2. Given reports that peri-vaccination use of antipyretic analgesics may attenuate vaccine immunogenicity (4), we also conducted an exploratory analysis to determine the influence of taking paracetamol or non-steroidal anti-inflammatory drugs (NSAIDs) to treat post-vaccination symptoms on post-vaccination antibody titres.

References

1. Cook AM, Faustini SE, Williams LJ, Cunningham AF, Drayson MT, Shields AM, et al. Validation of a combined ELISA to detect IgG, IgA and IgM antibody responses to SARS-CoV-2 in mild or moderate non-hospitalised patients. Journal of Immunological Methods. 2021;494:113046.
2. Holt H, Talaei M, Greenig M, Zenner D, Symons J, Relton C, et al. Risk factors for developing COVID-19: a population-based longitudinal study (COVIDENCE UK). Thorax. 2021.
3. Benjamini Y, Hochberg Y. Controlling the False Discovery Rate: A Practical and Powerful Approach to Multiple Testing. Journal of the Royal Statistical Society Series B (Methodological). 1995;57(1):289-300.
4. Saleh E, Moody MA, Walter EB. Effect of antipyretic analgesics on immune responses to vaccination. Hum Vaccin Immunother. 2016;12(9):2391-402.

Supplementary Tables

Table S1. Baseline questions

**Sociodemographic**	
Date of birth (DD/MM/YYYY)	
Post code	
Address	
Please state your **assigned sex at birth.**	• Male<br>• Female
What is your **current** height? (if you are unsure, please put your best estimate)	-Feet/inches<br>-Centimetres
What is your **current** weight?	- Stones (sts) / pounds (lbs)<br>- Kilograms (kg)
What is your ethnic origin?	• White<br>▪ English / Welsh / Scottish / Northern Irish / British<br>▪ Irish<br>▪ Gypsy or Irish Traveller<br>▪ Any other white background<br>• Mixed / Multiple ethnic groups<br>▪ White and Black Caribbean<br>▪ White and Black African<br>▪ White and Asian<br>▪ Any other Mixed / Multiple ethnic backgrounds<br>• Asian / Asian British<br>▪ Indian<br>▪ Pakistani<br>▪ Bangladeshi<br>▪ Chinese<br>▪ Any other Asian background<br>• Black / African / Caribbean / Black British<br>▪ African<br>▪ Caribbean<br>▪ Any other Black / African / Caribbean background<br>• Arab<br>• Other Ethnic Group
What is the highest level of education that you have completed?	• Primary school (0)<br>• Secondary school up to 16 years (1)<br>• Higher or secondary or further education (A-levels, BTEC, etc.) (2)<br>• College or university (3)<br>• Post-graduate degree (4)

Which of the following best describes your current occupational status?	• Employed<br>• Self-employed<br>• Retired<br>• Furloughed<br>• Unemployed<br>• Student<br>• Other
**Comorbidities**	
Have you ever been diagnosed with any of the following conditions by a doctor?<br><br>Select all that apply.	• Asthma<br>• Atopic Eczema or Atopic Dermatitis<br>• Autoimmune disease (e.g. rheumatoid arthritis, multiple sclerosis (MS), lupus (SLE), Crohn's disease, ulcerative colitis, psoriasis, Raynaud's disease, scleroderma)<br>• Cancer<br>• Cerebral Palsy<br>• COPD (including chronic bronchitis, and emphysema)<br>• Cystic Fibrosis<br>• Dementia<br>• Diabetes or pre-diabetes<br>• Hayfever or Allergic Rhinitis<br>• Heart Attack, Angina or Coronary Artery Disease<br>• Heart Failure<br>• High Blood Pressure (Hypertension)<br>• HIV Infection<br>• Hyperparathyroidism (overactive parathyroid gland)<br>• Kidney stones<br>• Other kidney disease<br>• Leg Artery Disease (also known as 'peripheral vascular disease', 'peripheral arterial disease' or 'intermittent claudication')<br>• Mental health disorder<br>• Motor Neurone Disease<br>• Organ transplant<br>• Parkinson's Disease<br>• Primary immune deficiency (e.g. antibody deficiency, combined immunodeficiency)<br>• Sarcoidosis<br>• Sickle Cell Disease (i.e. two copies of altered gene, affected by anaemia and other complications<br>• Sickle Cell Carrier (also known as 'sickle cell trait', with only one copy of altered gene: few symptoms if any)<br>• Splenectomy (removal of spleen)<br>• Stroke or Mini-Stroke<br>• Tuberculosis (TB)<br>• None of the above
You indicated you have been diagnosed with diabetes or pre-diabetes. Please specify your diagnosis:	• Pre-diabetes (high blood sugar levels, not enough to be diagnosed with diabetes)<br>• Type 1 diabetes<br>• Type 2 diabetes<br>• Other type of diabetes
Do you currently have cancer?	• Never<br>• No, cancer cured or in remission<br>• Yes, currently receiving treatment

Under each heading, please click the ONE box that best describes your health TODAY.<br>(Anxiety / Depression)	• I am not anxious or depressed<br>• I am moderately anxious or depressed<br>• I am extremely anxious or depressed
Over the last 12 months, would you say that on the whole, your health has been:	• Excellent<br>• Very good<br>• Good<br>• Fair<br>• Poor
**Vaccination**	
Have you ever had the BCG vaccine?<br><br>*This is the vaccine against Tuberculosis (TB), it's injected in the upper arm and usually leaves a small scar*	• Yes<br>• No<br>• Unsure
**Lifestyle**	
Which of these best describes your use of cigarettes?	• I have never smoked cigarettes<br>• I used to smoke cigarettes occasionally but now not at all<br>• I used to smoke cigarettes daily but now not at all<br>• I smoke cigarettes occasionally but not every day<br>• I smoke cigarettes daily
Which of these best describes your use of e-cigarettes (vaping)?	• I have never vaped or used e-cigarettes<br>• I used to use e-cigarettes occasionally, but now not at all<br>• I used to use e-cigarettes daily but now not at all<br>• I vape occasionally but not every day<br>• I vape daily
During the last week, roughly how many hours did you spend doing light exercise that does not make you particularly breathless, such as light gardening, walking, including walking for pleasure or exercise, walking to the shops, walking to work?	- 0 -10+ hours
During the last week, roughly how many hours did you spend doing more vigorous physical exercise of sufficient intensity to make you breathless or to raise your heart rate significantly, such as heavy physical work, more strenuous gardening (e.g. vigorous digging, landscaping) swimming, jogging, aerobics, football, tennis, cycling, gym workout?	- 0 -10+ hours
During the past month, how many hours of actual sleep did you get per night on average?<br>(This may be different than the number of hours you spent in bed)	• 0–24 hours
**Diet**	
How many units of alcohol did you drink over the last 7 days?<br>*One unit is ½ a pint (285 ml) of ordinary beer, lager or cider; 25ml of spirits; 1 small glass (75ml) of wine; or 50ml of sherry*	• None<br>• 1–7 units<br>• 8–14 units<br>• 15–21 units<br>• 22–28 units<br>• More than 28 units

Do you **exclude** any of the following foods from your diet? Select all that apply.	- Eggs<br>- Cow's milk or products made from cow's milk (e.g. cheese, yoghurt)<br>- Fish<br>- White meat (e.g. poultry)<br>- Red meat<br>- No, I eat all of these foods
Over the **last month**, have you taken any of the following supplements at least **once per week**?<br><br>Select all that apply.	• Multivitamin (including prenatal multivitamins)<br>• Supplement containing vitamin A only<br>• Supplement containing vitamin B only<br>• Supplement containing vitamin C only<br>• Supplement containing vitamin D only<br>• Supplement containing calcium only<br>• Supplement containing calcium and vitamin D combined<br>• Supplement containing vitamin E only<br>• Supplement containing zinc only<br>• Supplement containing iron only<br>• Supplement containing probiotics<br>• Supplement containing fish oil, krill oil or other source of omega-3 fatty acids<br>• Supplement containing cod liver oil<br>• Supplement containing echinacea<br>• Supplement containing garlic or garlic powder (allicin)<br>• Supplement containing turmeric / curcumin<br>• Supplement containing Cannabidiol (CBD) oil<br>• Supplements containing folic acid<br>• Supplement containing Selenium only<br>• Other (e.g. other micronutrients (such as herbal supplements) or combinations of micronutrients (such vitamin C & zinc)) Please specify:<br>• None of the above
**Medications**	

Please type the names of all the medications you are currently taking below, one medication per box. The next pages will collect details about dosage for each.

Include all types of medications taken at home or administered in a hospital or clinic (capsules, tablets, contraceptive pills or implants, inhalers, injections, intravenous infusions, monoclonal antibodies, chemotherapy, immunosuppressants, etc.)

Please note that other pages will collect details about the amount of medicine in each dose (next page) and how often you take each dose (the page after that). If there are any details about your medication that aren't captured by our form (e.g. if you take different doses of a medicine at different times of day), there will be space to enter them in a blank text box at the end of this section of the questionnaire.

	**Medication name (generic or brand name, either is fine)**
Medication 1	
Medication 2	
….	

Table S2. Monthly follow-up questions

**Questions**	
Since you last checked in with us, have you had a nose or throat swab for COVID-19 or any other respiratory virus, or has a result from a previous swab test become newly available?<br>(This question is about tests to detect the virus itself: they are usually done in somebody who has symptoms, but screening of asymptomatic people can also be done. It's usually a nose/throat swab, but saliva tests are also becoming available)	• Yes<br>• No
On what date did you have this nose / throat swab?<br>If you are not sure of the exact date, enter the approximate date (DD/MM/YYYY).	
What was the result? Click as many as apply.	• Positive for COVID-19 (SARS-CoV-2 coronavirus)<br>• Positive for influenza virus<br>• Positive for another respiratory virus<br>• Negative for all/any viruses tested<br>• Not known
Since you last checked in with us, have you had an ANTIBODY test for COVID-19? (This is a test to detect whether or not you have already had the virus, usually done in somebody who does not currently have symptoms. It's usually a blood test / fingerprick test, but saliva tests are also available)	• Yes<br>• No
On what date did you have this ANTIBODY test? If you are not sure of the exact date, enter the approximate date (DD/MM/YYYY)	
What was the result?	• Positive<br>• Negative<br>• Not known
Since you last checked in with us, have you had a vaccine (immunisation) against COVID-19?	• Yes<br>• No
On what date did you have this COVID-19 vaccine (immunisation)? If you are not sure of the exact date, enter the approximate date (DD/MM/YYYY)	
Since you last checked in with us (last questionnaire sent <previous monthly questionnaire date>), have you had one or more doses of a COVID-19 vaccine (immunisation)? First doses and booster doses both count.	• Yes, I have had one or more doses of COVID-19 vaccine <strong>since I filled my last questionnaire<br>• No, I have not had any doses of COVID-19 vaccine <strong>since I filled my last questionnaire<br>• Not sure (e.g. you took part in vaccine trial, but dont yet know whether or not you had the real vaccine or the placebo (dummy vaccine)
How many doses of COVID-19 vaccine have you had since you last checked in with us? (last questionnaire sent 18th November 2021)	• One dose since my last questionnaire<br>• Two doses since my last questionnaire
On what date did you have the first of these vaccine doses? If you are not sure of the exact date, enter the approximate date (DD/MM/YYYY) e.g. for 16th December 2020, write 16/12/2020	

On what date did you have the second of these vaccine doses? If you are not sure of the exact date, enter the approximate date (DD/MM/YYYY) e.g. for 16th December 2020, write 16/12/2020	
Which COVID-19 vaccine did you have?	• Oxford / AstraZeneca / ChAdOx1<br>• Pfizer / BioNTech<br>• Moderna<br>• Valneva<br>• Novavax<br>• Janssen (also known as Johnson & Johnson)<br>• Other - please specify<br>• Not sure / don't know
Did you experience any of the following symptoms following your COVID-19 vaccine?	• Tenderness, soreness, swelling, redness or a painful, heavy feeling at the injection site<br>• Feeling tired<br>• Headache<br>• Fever / high temperature (37.8° C or greater)<br>• Muscle aches<br>• Swelling of the glands in your armpit or neck<br>• Other symptoms: specify<br>• None of the above
Did you take any of the following to treat post-vaccination symptoms (e.g. fever, muscle aches etc)?	• Paracetamol (e.g. Disprol, Hedex, Medinol, Panadol)<br>• Ibuprofen (e.g. Nurofen, Brufen, Calprofen)<br>• Something else: please write name here<br>• None of the above
Since you last checked in with us, have you experienced any of the following symptoms: cold or flu symptoms, sore throat, persistent cough, loss of smell or taste, fever, fatigue, diarrhoea, abdominal pain or loss of appetite?	• Yes, I have had one or more of these symptoms since completing my last COVIDENCE UK questionnaire<br>• No, I have not had any of these symptoms since completing my last COVIDENCE UK questionnaire
When did your symptoms start? (DD/MM/YYYY)	
Did you have a persistent cough (coughing a lot for more than an hour, or 3 or more coughing episodes in 24 hours)?	• No<br>• Persistent dry cough (i.e. producing little or no phlegm)<br>• Persistent productive cough
Did you experience unusual fatigue?	• No<br>• Mild fatigue<br>Severe fatigue – I struggled to get out of bed
Did you have a loss of sense of smell or taste?	• Yes<br>• No
Did you skip any meals because you felt unwell?	• Yes<br>• No

Table S3. Determinants of antibody titres following administration of two doses of ChAdOx1 nCoV-19 vaccine.

Predictor	N Seropositive (%)	Median titre, U/ml (IQR)	Minimally Adjusted % difference (95% CI) [1]	P value	Fully Adjusted % difference (95% CI) [2]	P value
Vaccine timing						
Time since fully vaccinated, wks						
<2	17/5436 (0.3)	2.31 (1.48, 3.08)	-8.14 (-27.78, 16.85)	0.49	-12.25 (-31.35, 12.17)	0.30
2-4	513/5436 (9.4)	2.71 (2.01, 3.80)	Referent			
5-8	2924/5436 (53.8)	2.45 (1.80, 3.35)	-7.99 (-12.27, -3.51)	0.001	-8.39 (-12.64, -3.95)	<0.001*
9-16	1890/5436 (34.8)	2.22 (1.68, 2.99)	-14.78 (-19, -10.34)	<0.001	-13.67 (-18, -9.1)	<0.001*
>16	92/5436 (1.7)	2.39 (1.66, 3.77)	-9.11 (-18.69, 1.6)	0.09	3.04 (-9.36, 17.13)	0.65
Inter-vaccine interval, weeks						
<6	110/5436 (2.0)	1.92 (1.38, 2.98)	-14.8 (-22.5, -6.34)	0.001	-10.2 (-19.66, 0.38)	0.06
6-10	1731/5436 (31.8)	2.34 (1.71, 3.17)	-6.27 (-8.94, -3.52)	<0.001	-4.36 (-7.25, -1.38)	0.004*
>10	3595/5436 (66.1)	2.44 (1.81, 3.35)	Referent			
Time of second vaccination						
Morning	2291/5254 (43.6)	2.38 (1.76, 3.27)	Referent			
Lunchtime	930/5254 (17.7)	2.35 (1.75, 3.18)	-1.93 (-5.6, 1.88)	0.32		
Afternoon	1500/5254 (28.5)	2.43 (1.78, 3.36)	1.96 (-1.3, 5.34)	0.24		
Evening	533/5254 (10.1)	2.42 (1.78, 3.36)	0.21 (-4.42, 5.06)	0.93		
Quarter of second vaccination						
Q1	217/5436 (4.0)	2.08 (1.66, 2.91)	-9.84 (-15.77, -3.49)	0.003	-6.31 (-14.47, 2.62)	0.16
Q2	5204/5436 (95.7)	2.41 (1.78, 3.31)	Referent			
Q3	9/5436 (0.2)	1.38 (1.36, 3.24)	-13.65 (-37.7, 19.68)	0.38	33.63 (-10.87, 100.34)	0.16
Q4	6/5436 (0.1)	1.28 (1.22, 1.49)	-44.55 (-62.82, -17.3)	0.004	-72.2 (-85.48, -46.78)	<0.001*
Socio-demographic factors (baseline)						
Age						
<30	27/5436 (0.5)	2.34 (1.60, 3.69)	Referent			
30-39.99	92/5436 (1.7)	2.42 (1.79, 3.15)	2.02 (-17.61, 26.34)	0.85		
40-49.99	350/5436 (6.4)	2.47 (1.88, 3.51)	9.83 (-9.63, 33.49)	0.35		
50-59.99	1519/5436 (27.9)	2.61 (1.91, 3.59)	14.13 (-5.58, 37.96)	0.17		
60-69.99	2358/5436 (43.4)	2.37 (1.75, 3.21)	2.65 (-15.03, 24.01)	0.79		
70-79.99	1052/5436 (19.4)	2.18 (1.64, 2.97)	-4.22 (-20.83, 15.88)	0.66		
≥80	38/5436 (0.7)	1.83 (1.39, 2.44)	-16.61 (-34.79, 6.65)	0.15		
Sex						
Female	3895/5436 (71.7)	2.41 (1.79, 3.32)	Referent			
Male	1541/5436 (28.3)	2.34 (1.71, 3.21)	-1.66 (-4.54, 1.32)	0.27		

Predictor	N Seropositive (%)	Median titre, U/ml (IQR)	Minimally Adjusted % difference (95% CI) [1]	P value	Fully Adjusted % difference (95% CI) [2]	P value
Ethnicity						
White	5236/5436 (96.3)	2.38 (1.76, 3.26)	Referent			
Mixed/Multiple/Other ethnic group	116/5436 (2.1)	2.67 (1.97, 3.73)	17.1 (6.83, 28.36)	0.001	10.19 (-0.01, 21.44)	0.05
South Asian	61/5436 (1.1)	3.15 (2.22, 4.06)	20.78 (6.46, 37.02)	0.003	21.23 (4.64, 40.45)	0.010*
Black/African/Caribbean/Black British	23/5436 (0.4)	2.98 (2.20, 5.12)	27.16 (3.65, 56.01)	0.021	10.44 (-11.26, 37.43)	0.37
BMI, kg/m2						
<25	2702/5427 (49.8)	2.38 (1.77, 3.20)	Referent			
25-30	1758/5427 (32.4)	2.38 (1.76, 3.28)	2.74 (-0.31, 5.88)	0.079	1.96 (-1.22, 5.24)	0.23
>30	967/5427 (17.8)	2.49 (1.74, 3.61)	4.68 (0.90, 8.60)	0.015	3.63 (-0.54, 7.97)	0.09
Highest educational level attained						
Primary/Secondary	587/5435 (10.8)	2.34 (1.66, 3.30)	3.62 (-1.16, 8.63)	0.14	0.98 (-3.97, 6.18)	0.70
Higher/further (A levels)	805/5435 (14.8)	2.52 (1.83, 3.52)	7.08 (2.66, 11.69)	0.001	3.28 (-1.17, 7.93)	0.15
College	2409/5435 (44.3)	2.43 (1.80, 3.32)	4.76 (1.53, 8.10)	0.004	3.99 (0.64, 7.45)	0.019*
Post-grad	1634/5435 (30.1)	2.34 (1.73, 3.17)	Referent			
Quantiles of IMD rank						
Q1 (least wealthy)	1093/5431 (20.1)	2.43 (1.78, 3.42)	1.97 (-1.90, 6.00)	0.35		
Q2	1335/5431 (24.6)	2.36 (1.73, 3.22)	-1.49 (-5.03, 2.18)	0.45		
Q3	1452/5431 (26.7)	2.39 (1.79, 3.28)	-0.11 (-3.62, 3.53)	0.95		
Q4 (most wealthy)	1551/5431 (28.6)	2.39 (1.77, 3.28)	Referent			
Lifestyle factors						
Tobacco smoking						
No	5223/5436 (96.1)	2.39 (1.76, 3.29)	Referent			
Yes	213/5436 (3.9)	2.52 (1.88, 3.42)	0.77 (-5.92, 7.94)	0.83		
Vaping						
No	5304/5422 (97.8)	2.39 (1.76, 3.29)	Referent			
Yes	118/5422 (2.2)	2.65 (1.92, 3.63)	7.87 (-1.55, 18.20)	0.10		
Alcohol, units/wk						
None	1403/5436 (25.8)	2.42 (1.77, 3.26)	Referent			
1-7	1924/5436 (35.4)	2.41 (1.79, 3.35)	1.75 (-1.69, 5.31)	0.32		
8-14	1113/5436 (20.5)	2.33 (1.73, 3.25)	0.70 (-3.20, 4.75)	0.73		
15-21	554/5436 (10.2)	2.40 (1.76, 3.32)	2.50 (-2.44, 7.69)	0.33		
22-28	249/5436 (4.6)	2.22 (1.73, 2.96)	-3.95 (-10.25, 2.80)	0.25		
>28	193/5436 (3.6)	2.48 (1.82, 3.45)	3.38 (-4.16, 11.52)	0.39		
Light exercise, hrs/wk						
0-4	1643/5420 (30.3)	2.47 (1.81, 3.45)	3.91 (0.52, 7.42)	0.024	1.78 (-1.76, 5.45)	0.33
5-9	1785/5420 (32.9)	2.37 (1.73, 3.27)	0.82 (-2.36, 4.10)	0.62	-0.54 (-3.84, 2.88)	0.76
≥10	1992/5420 (36.8)	2.36 (1.76, 3.19)	Referent			

Predictor	N Seropositive (%)	Median titre, U/ml (IQR)	Minimally Adjusted % difference (95% CI) [1]	P value	Fully Adjusted % difference (95% CI) [2]	P value
Vigorous exercise, hrs/wk						
0	2032/5420 (37.5)	2.42 (1.76, 3.39)	4.87 (1.33, 8.54)	0.007	1.76 (-2.01, 5.67)	0.37
1-3	2013/5420 (37.1)	2.39 (1.78, 3.28)	2.91 (-0.57, 6.51)	0.10	1.08 (-2.47, 4.77)	0.56
≥4	1375/5420 (25.4)	2.36 (1.73, 3.15)	Referent			
Sleep, hrs/night						
≤5	468/5433 (8.6)	2.52 (1.88, 3.48)	4.83 (-0.51, 10.44)	0.099	3.44 (-2.18, 9.39)	0.24
6	1301/5433 (23.9)	2.37 (1.78, 3.31)	-0.42 (-4.09, 3.40)	0.74	-1.37 (-5.18, 2.58)	0.49
7	2242/5433 (41.3)	2.37 (1.73, 3.24)	-2.31 (-5.50, 0.99)	0.19	-1.66 (-4.99, 1.79)	0.34
≥8	1422/5433 (26.2)	2.41 (1.77, 3.32)	Referent			
Self-assessed general health						
Excellent	1134/5436 (20.9)	2.38 (1.78, 3.23)	Referent			
Very good	2215/5436 (40.7)	2.37 (1.73, 3.24)	0.38 (-3.15, 4.04)	0.84	-0.18 (-3.82, 3.6)	0.93
Good	1350/5436 (24.8)	2.44 (1.82, 3.37)	4.25 (0.21, 8.45)	0.039	2.48 (-1.83, 6.98)	0.26
Fair	584/5436 (10.7)	2.43 (1.77, 3.35)	1.37 (-3.58, 6.56)	0.6	0.97 (-4.54, 6.79)	0.74
Poor	153/5436 (2.8)	2.38 (1.70, 3.65)	4.91 (-3.59, 14.16)	0.27	-2.58 (-11.87, 7.69)	0.61
Anxiety or depression						
No	4096/5433 (75.4)	2.39 (1.76, 3.27)	Referent			
Yes	1337/5433 (24.6)	2.41 (1.77, 3.35)	0.08 (-3.00, 3.26)	0.96		
Food choice						
None	5123/5436 (94.2)	2.40 (1.77, 3.30)	Referent			
Vegetarian	237/5436 (4.4)	2.34 (1.73, 3.31)	-3.42 (-9.52, 3.10)	0.30		
Vegan	76/5436 (1.4)	2.33 (1.74, 2.95)	-6.65 (-16.65, 4.55)	0.23		
Medical conditions						
Heart disease [3]						
No	5236/5436 (96.3)	2.40 (1.77, 3.31)	Referent			
Yes	200/5436 (3.7)	2.15 (1.56, 2.75)	-5.04 (-11.61, 2.02)	0.16		
Arterial disease [4]						
No	5169/5436 (95.1)	2.40 (1.77, 3.31)	Referent			
Yes	267/5436 (4.9)	2.20 (1.59, 2.93)	-4.11 (-9.93, 2.08)	0.19		
Hypertension						
No	4198/5436 (77.2)	2.42 (1.80, 3.32)	Referent			
Yes	1238/5436 (22.8)	2.32 (1.68, 3.20)	-1.39 (-4.55, 1.87)	0.4		
Immunodeficiency [5]						
No	5412/5436 (99.6)	2.40 (1.77, 3.30)	Referent			
Yes	24/5436 (0.4)	1.95 (1.61, 2.67)	-14.47 (-30.00, 4.52)	0.13		
Major neurological conditions [6]						
No	5304/5436 (97.6)	2.40 (1.77, 3.31)	Referent			

Predictor	N Seropositive (%)	Median titre, U/ml (IQR)	Minimally Adjusted % difference (95% CI) [1]	P value	Fully Adjusted % difference (95% CI) [2]	P value
Yes	132/5436 (2.4)	1.98 (1.56, 2.88)	-9.84 (-17.31, -1.69)	0.02	-8.84 (-16.78, -0.14)	0.047
Cancer						
Never	4899/5436 (90.1)	2.40 (1.78, 3.31)	Referent			
Past (cured or in remission)	497/5436 (9.1)	2.34 (1.71, 3.22)	-0.62 (-5.13, 4.10)	0.79	-0.73 (-5.4, 4.18)	0.77
Present (active)	40/5436 (0.7)	1.90 (1.46, 2.76)	-15.79 (-27.93, -1.61)	0.03	-15.51 (-29.02, 0.59)	0.06
Asthma						
No	4556/5436 (83.8)	2.38 (1.75, 3.29)	Referent			
Yes	880/5436 (16.2)	2.46 (1.83, 3.41)	1.74 (-1.87, 5.49)	0.35		
COPD						
No	5335/5436 (98.1)	2.40 (1.76, 3.29)	Referent			
Yes	101/5436 (1.9)	2.37 (1.77, 3.70)	3.78 (-5.96, 14.53)	0.46		
Diabetes types						
No diabetes	5028/5430 (92.6)	2.40 (1.78, 3.30)	Referent			
Pre-diabetes	181/5430 (3.3)	2.34 (1.71, 3.27)	0.41 (-6.78, 8.16)	0.91		
Type 1 diabetes	33/5430 (0.6)	2.29 (1.68, 3.17)	-3.87 (-19.00, 14.08)	0.65		
Type 2 diabetes	188/5430 (3.5)	2.14 (1.46, 3.14)	-4.14 (-10.9, 3.13)	0.26		
Atopy [7]						
No	4048/5436 (74.5)	2.38 (1.76, 3.26)	Referent			
Yes	1388/5436 (25.5)	2.42 (1.80, 3.35)	0.82 (-2.23, 3.95)	0.6		
Pre-vaccination SARS-COV-2 infection/COVID-19						
Seronegative	3491/4150 (84.1)	2.29 (1.72, 3.02)	Referent			
Seropositive, asymptomatic	489/4150 (11.8)	3.08 (2.16, 4.43)	40.16 (34.16, 46.42)	<0.001	39.83 (33.87, 46.05)	<0.001*
Seropositive, symptomatic	170/4150 (4.1)	5.02 (3.76, 8.23)	120.94 (106.67, 136.19)	<0.001	119.95 (105.74, 135.14)	<0.001*
Nutritional supplements						
Multivitamin						
No	4298/5436 (79.1)	2.40 (1.76, 3.31)	Referent			
Yes	1138/5436 (20.9)	2.38 (1.78, 3.23)	-1.97 (-5.13, 1.29)	0.23		
Vitamin A						
No	5407/5436 (99.5)	2.39 (1.76, 3.30)	Referent			
Yes	29/5436 (0.5)	2.18 (1.73, 3.37)	-8.97 (-24.15, 9.24)	0.31		
Vitamin C						
No	4887/5436 (89.9)	2.38 (1.76, 3.28)	Referent			
Yes	549/5436 (10.1)	2.49 (1.81, 3.49)	5.78 (1.22, 10.55)	0.01	3.28 (-1.45, 8.22)	0.18
Vitamin D						
No	2642/5436 (48.6)	2.40 (1.78, 3.30)	Referent			

Predictor	N Seropositive (%)	Median titre, U/ml (IQR)	Minimally Adjusted % difference (95% CI) [1]	P value	Fully Adjusted % difference (95% CI) [2]	P value
Yes	2794/5436 (51.4)	2.39 (1.75, 3.29)	-0.82 (-3.43, 1.86)	0.54		
Zinc						
No	5189/5436 (95.5)	2.40 (1.76, 3.30)	Referent			
Yes	247/5436 (4.5)	2.32 (1.78, 3.16)	-3.19 (-9.17, 3.19)	0.32		
Selenium						
No	5375/5436 (98.9)	2.40 (1.77, 3.30)	Referent			
Yes	61/5436 (1.1)	2.19 (1.55, 2.99)	-12.98 (-23.29, -1.28)	0.03	-16.87 (-27.57, -4.58)	0.009*
Iron						
No	5255/5436 (96.7)	2.40 (1.77, 3.30)	Referent			
Yes	181/5436 (3.3)	2.19 (1.74, 3.18)	-4.79 (-11.60, 2.55)	0.20		
Probiotics						
No	5078/5436 (93.4)	2.39 (1.76, 3.30)	Referent			
Yes	358/5436 (6.6)	2.44 (1.83, 3.26)	-0.01 (-5.24, 5.52)	1.0		
Omega-3						
No	4765/5436 (87.7)	2.39 (1.75, 3.29)	Referent			
Yes	671/5436 (12.3)	2.42 (1.85, 3.33)	3.04 (-1.04, 7.29)	0.15		
Cod liver oil						
No	4951/5436 (91.1)	2.40 (1.77, 3.30)	Referent			
Yes	485/5436 (8.9)	2.32 (1.70, 3.17)	-0.86 (-5.40, 3.89)	0.72		
Garlic						
No	5312/5436 (97.7)	2.39 (1.76, 3.29)	Referent			
Yes	124/5436 (2.3)	2.60 (1.86, 3.58)	10.33 (0.92, 20.60)	0.03	15.18 (5.13, 26.2)	0.002*
Medications						
Beta-2 adrenergic agonists						
No	4948/5436 (91.0)	2.39 (1.76, 3.29)	Referent			
Yes	488/5436 (9.0)	2.49 (1.81, 3.46)	2.60 (-2.07, 7.49)	0.28		
Beta blockers						
No	5057/5436 (93.0)	2.40 (1.78, 3.31)	Referent			
Yes	379/5436 (7.0)	2.29 (1.62, 3.05)	-4.57 (-9.45, 0.58)	0.08	-1.95 (-7.33, 3.74)	0.49
Statins						
No	4513/5436 (83.0)	2.43 (1.80, 3.34)	Referent			
Yes	923/5436 (17.0)	2.18 (1.61, 2.95)	-6.05 (-9.49, -2.48)	0.001	-6.57 (-10.24, -2.75)	0.001*
ACE inhibitors						
No	4906/5436 (90.3)	2.41 (1.79, 3.31)	Referent			
Yes	530/5436 (9.7)	2.18 (1.60, 3.08)	-5.62 (-9.81, -1.24)	0.01	-5.3 (-9.82, -0.55)	0.029*
Proton pump inhibitors						
No	4694/5436 (86.4)	2.41 (1.78, 3.31)	Referent			

Predictor	N Seropositive (%)	Median titre, U/ml (IQR)	Minimally Adjusted % difference (95% CI) [1]	P value	Fully Adjusted % difference (95% CI) [2]	P value
Yes	742/5436 (13.6)	2.26 (1.66, 3.09)	-3.83 (-7.50, -0.02)	0.05	-6.33 (-10.25, -2.24)	0.003*
H2-receptor antagonists						
No	5399/5436 (99.3)	2.39 (1.76, 3.30)	Referent			
Yes	37/5436 (0.7)	2.38 (1.73, 3.17)	3.45 (-11.98, 21.60)	0.68		
Inhaled corticosteroids						
No	5087/5436 (93.6)	2.40 (1.76, 3.31)	Referent			
Yes	349/5436 (6.4)	2.38 (1.79, 3.15)	-2.38 (-7.53, 3.06)	0.38		
Bronchodilators						
No	4930/5436 (90.7)	2.38 (1.76, 3.28)	Referent			
Yes	506/5436 (9.3)	2.51 (1.82, 3.45)	3.27 (-1.35, 8.11)	0.17		
Systemic Immunosuppressants						
No	5185/5436 (95.4)	2.40 (1.76, 3.31)	Referent			
Yes	251/5436 (4.6)	2.36 (1.79, 3.14)	-2.07 (-8.08, 4.33)	0.52		
Angiotensin receptor blockers						
No	5084/5436 (93.5)	2.39 (1.77, 3.29)	Referent			
Yes	352/5436 (6.5)	2.39 (1.67, 3.39)	3.46 (-2.01, 9.25)	0.22		
Selective serotonin reuptake inhibitors						
No	5104/5436 (93.9)	2.39 (1.76, 3.28)	Referent			
Yes	332/5436 (6.1)	2.47 (1.77, 3.60)	4.24 (-1.41, 10.22)	0.14		
non-SSRIs antidepressants						
No	5218/5436 (96.0)	2.40 (1.76, 3.29)	Referent			
Yes	218/5436 (4.0)	2.34 (1.74, 3.43)	1.75 (-4.92, 8.89)	0.62		
Calcium channel blockers						
No	4870/5436 (89.6)	2.40 (1.78, 3.31)	Referent			
Yes	566/5436 (10.4)	2.29 (1.65, 3.17)	-1.92 (-6.17, 2.52)	0.39		
Thiazides						
No	5235/5436 (96.3)	2.40 (1.77, 3.31)	Referent			
Yes	201/5436 (3.7)	2.22 (1.61, 3.04)	-4.71 (-11.23, 2.29)	0.18		
Vitamin K antagonists						
No	5399/5436 (99.3)	2.39 (1.77, 3.30)	Referent			
Yes	37/5436 (0.7)	2.13 (1.72, 3.22)	-2.44 (-17.03, 14.72)	0.77		
Sodium-glucose co-transporter-2 inhibitors						
No	5414/5436 (99.6)	2.39 (1.77, 3.30)	Referent			
Yes	22/5436 (0.4)	2.56 (1.43, 4.23)	-1.18 (-19.84, 21.83)	0.91		
Anticholinergics						
No	5192/5436 (95.5)	2.39 (1.76, 3.29)	Referent			
Yes	244/5436 (4.5)	2.42 (1.81, 3.32)	0.81 (-5.46, 7.50)	0.8		

Predictor	N Seropositive (%)	Median titre, U/ml (IQR)	Minimally Adjusted % difference (95% CI) [1]	P value	Fully Adjusted % difference (95% CI) [2]	P value
Metformin						
No	5301/5436 (97.5)	2.39 (1.77, 3.29)	Referent			
Yes	135/5436 (2.5)	2.44 (1.54, 3.69)	2.58 (-5.84, 11.75)	0.56		
Bisphosphonates						
No	5331/5436 (98.1)	2.39 (1.76, 3.30)	Referent			
Yes	105/5436 (1.9)	2.36 (1.79, 3.31)	2.67 (-6.81, 13.12)	0.59		
Anti-platelet drugs						
No	5101/5436 (93.8)	2.40 (1.78, 3.31)	Referent			
Yes	335/5436 (6.2)	2.24 (1.58, 3.05)	-4.14 (-9.38, 1.40)	0.14		
Sex hormone therapy						
No	4995/5436 (91.9)	2.39 (1.76, 3.29)	Referent			
Yes	441/5436 (8.1)	2.41 (1.82, 3.33)	-0.31 (-5.12, 4.75)	0.9		
Aspirin [8]						
No	5168/5436 (95.1)	2.40 (1.77, 3.31)	Referent			
Yes	268/5436 (4.9)	2.22 (1.59, 3.02)	-3.69 (-9.50, 2.50)	0.24		
Paracetamol [8]						
No	5211/5436 (95.9)	2.40 (1.77, 3.30)	Referent			
Yes	225/5436 (4.1)	2.33 (1.73, 3.31)	-0.31 (-6.75, 6.57)	0.93		
BCG vaccinated						
No	633/5427 (11.7)	2.38 (1.79, 3.13)	Referent			
Yes	4285/5427 (79.0)	2.41 (1.76, 3.32)	2.09 (-2.09, 6.44)	0.91		

Abbreviations: IQR, inter quartile range; CI, confidence interval; BMI, body mass index; IMD, index of multiple deprivation; COPD, chronic obstructive pulmonary disease; SARS-COV-2, severe acute respiratory syndrome coronavirus 2; COVID-19, coronavirus disease 2019; ACE, angiotensin-converting enzyme; H2, histamine 2; SSRIs, selective serotonin reuptake inhibitors; BCG, bacillus calmette-guérin

(1) Adjusted for age and sex only
(2) Adjusted for age, sex, Time since fully vaccinated, inter-dose interval, quarter of second vaccination, ethnicity, BMI, level of education, light exercise, vigorous exercise, sleep, self-assessed general health, major neurological condition, cancer, pre-vaccination SARS-CoV-2 status, use of vitamin C supplements, selenium supplements, garlic supplements, beta blockers, statins, ACE inhibitors, and proton pump inhibitors.
(3) Heart disease defined as coronary artery disease or heart failure
(4) Arterial disease defined as ischaemic heart disease, peripheral vascular disease or cerebrovascular disease
(5) Immunodeficiency defined as HIV, primary immune deficiency or other immunodeficiency
(6) Major neurological conditions defined as stroke, transient ischaemic attack, dementia, Parkinson's disease, multiple sclerosis or motor neuron disease
(7) Atopy defined as atopic eczema/dermatitis and/or hayfever/allergic rhinitis
(8) Chronic use prior to vaccination (i.e. distinct from acute post-vaccination use for treatment of reactogenic symptoms)

* Below multiple comparisons testing critical p-value threshold of 0.033

Table S4. Determinants of antibody titres following administration of two doses of BNT162b2 vaccine.

Predictor	N Seropositive (%)	Median titre, U/ml (IQR)	Minimally Adjusted % difference (95% CI) [1]	P value	Fully Adjusted % difference (95% CI) [2]	P value
Vaccine timing						
Time since fully vaccinated, wks						
<2	14/3291 (0.4)	10.56 (2.96, 14.2	46.59 (9.91, 95.50)	0.009	58.19 (3.5, 141.77)	0.034
2-4	61/3291 (1.9)	4.35 (3.32, 5.98)	Referent			
5-8	827/3291 (25.1)	4.20 (3.55, 4.96)	-1.85 (-13.72, 11.66)	0.78	-10.27 (-22.68, 4.14)	0.15
9-16	2052/3291 (62.4)	3.89 (3.14, 4.82)	-13.01 (-23.33, -1.30)	0.031	-17.34 (-28.73, -4.14)	0.012*
>16	337/3291 (10.2)	3.26 (2.21, 4.59)	-29.31 (-38.25, -19.07)	<0.001	-19.76 (-32.85, -4.12)	0.025*
Inter-vaccine interval, weeks						
<6	339/3291 (10.3)	3.21 (2.32, 4.35)	-23.39 (-27.7, -18.84)	<0.001	-12.28 (-20.34, -3.41)	0.008*
6-10	1086/3291 (33.0)	3.85 (3.10, 4.75)	-10.29 (-13.6, -6.86)	<0.001	-6.32 (-10.67, -1.75)	0.007*
>10	1866/3291 (56.7)	4.11 (3.35, 5.00)	Referent			
Time of second vaccination						
Morning	1253/3158 (39.7)	4.00 (3.22, 4.87)	Referent			
Lunchtime	557/3158 (17.6)	3.97 (3.21, 4.84)	-2.72 (-7.48, 2.27)	0.28	-7.72 (3.59, -55.5)	0.45
Afternoon	970/3158 (30.7)	4.00 (3.10, 4.90)	-1.14 (-5.21, 3.11)	0.59	-5.31 (4.19, -21.9)	0.78
Evening	378/3158 (12.0)	3.76 (2.90, 4.80)	-6.75 (-11.99, -1.2)	0.018	-9.37 (3.96, -60.5)	0.40
Quarter of second vaccination						
Q1	1045/3291 (31.8)	3.66 (2.79, 4.63)	-14.33 (-17.43, -11.11)	<0.001	-7.12 (-12.84, -1.03)	0.023*
Q2	2238/3291 (68.0)	4.09 (3.33, 4.97)	Referent			
Q3	2/3291 (0.1)	13.03 (4.47, 21.5	126.62 (13.39, 352.89)	0.021	-	
Q4	6/3291 (0.2)	3.24 (2.88, 4.32)	-17.43 (-44.67, 23.23)	0.35	36.15 (-44.77, 235.66)	0.50
Socio-demographic factors						
Age						
<30	53/3291 (1.6)	4.26 (3.50, 6.09)	Referent			
30-39.99	94/3291 (2.9)	4.44 (3.60, 5.28)	-7 (-21.5, 10.18)	0.4	-14.01 (-31.27, 7.58)	0.19
40-49.99	234/3291 (7.1)	4.05 (3.29, 5.11)	-12.82 (-24.97, 1.3)	0.073	-15.54 (-31, 3.38)	0.10
50-59.99	685/3291 (20.8)	4.18 (3.40, 5.19)	-8.3 (-20.33, 5.55)	0.227	-13.81 (-28.73, 4.23)	0.13
60-69.99	1196/3291 (36.3)	3.99 (3.23, 4.87)	-13.62 (-24.79, -0.78)	0.038	-19.3 (-33.18, -2.55)	0.026*
70-79.99	894/3291 (27.2)	3.76 (3.00, 4.59)	-19.33 (-29.85, -7.24)	0.003	-23.05 (-36.36, -6.95)	0.007*
≥80	135/3291 (4.1)	3.08 (2.34, 3.89)	-34.11 (-43.89, -22.63)	<0.001	-33.63 (-48.51, -14.46)	0.002*
Sex						
Female	2345/3291 (71.3)	4.03 (3.23, 4.95)	Referent			
Male	946/3291 (28.7)	3.82 (2.92, 4.61)	-4.80 (-8.46, -0.99)	0.01	-2.53 (-6.95, 2.09)	0.28

Predictor	N Seropositive (%)	Median titre, U/ml (IQR)	Minimally Adjusted % difference (95% CI) [1]	P value	Fully Adjusted % difference (95% CI) [2]	P value
Ethnicity						
White	3183/3290 (96.7)	3.95 (3.13, 4.84)	Referent			
Mixed/Multiple/Other ethnic group	59/3290 (1.8)	4.49 (3.66, 5.71)	9.38 (-4.02, 24.66)	0.18	9.48 (-6.79, 28.6)	0.27
South Asian	38/3290 (1.2)	4.04 (3.42, 5.25)	6.03 (-9.82, 24.67)	0.48	14.34 (-5.9, 38.94)	0.18
Black/African/Caribbean/Black British	10/3290 (0.3)	5.01 (3.91, 7.15)	38.93 (1.57, 90.04)	0.040	13.34 (-21.34, 63.3)	0.50
BMI, kg/m2						
<25	1575/3289 (47.9)	3.89 (3.11, 4.85)	Referent			
25-30	1084/3289 (33.0)	3.98 (3.18, 4.87)	3.28 (-0.69, 7.41)	0.11		
>30	630/3289 (19.2)	4.10 (3.22, 4.86)	1.01 (-3.61, 5.84)	0.67		
Highest educational level attained						
Primary/Secondary	396/3286 (12.1)	4.03 (3.18, 4.94)	4.71 (-1.37, 11.17)	0.13		
Higher/further (A levels)	437/3286 (13.3)	3.90 (3.12, 4.84)	0.84 (-4.78, 6.78)	0.78		
College	1481/3286 (45.1)	3.95 (3.16, 4.88)	1.34 (-2.73, 5.59)	0.52		
Post-grad	972/3286 (29.6)	3.99 (3.15, 4.84)	Referent			
Quantiles of IMD rank						
Q1 (least wealthy)	778/3289 (23.7)	3.89 (3.04, 4.87)	-4.67 (-9.22, 0.1)	0.055	-2.74 (-8.26, 3.1)	0.35
Q2	772/3289 (23.5)	4.09 (3.36, 4.93)	3.32 (-1.58, 8.48)	0.19	2.3 (-3.37, 8.3)	0.44
Q3	857/3289 (26.1)	3.84 (3.06, 4.68)	-4.43 (-8.85, 0.21)	0.061	-4.93 (-10.01, 0.43)	0.07
Q4 (most wealthy)	882/3289 (26.8)	4.02 (3.17, 4.94)	Referent			
Lifestyle factors						
Tobacco smoking						
No	3154/3291 (95.8)	3.97 (3.15, 4.86)	Referent			
Yes	137/3291 (4.2)	3.82 (3.25, 4.88)	-3.06 (-11.10, 5.71)	0.48		
Vaping						
No	3213/3282 (97.9)	3.96 (3.15, 4.86)	Referent			
Yes	69/3282 (2.1)	4.13 (3.42, 4.94)	-1.84 (-13.01, 10.76)	0.76		
Alcohol, units/wk						
None	880/3291 (26.7)	4.05 (3.24, 5.02)	Referent			
1-7	1203/3291 (36.6)	3.95 (3.15, 4.85)	-3.67 (-7.81, 0.65)	0.095	-1.85 (-6.76, 3.32)	0.48
8-14	684/3291 (20.8)	3.97 (3.14, 4.85)	-4.4 (-9.11, 0.55)	0.081	-1.63 (-7.29, 4.37)	0.59
15-21	309/3291 (9.4)	3.74 (3.07, 4.61)	-7.85 (-13.72, -1.58)	0.015	-3.42 (-10.54, 4.26)	0.37
22-28	124/3291 (3.8)	4.04 (3.16, 4.69)	-2.72 (-11.58, 7.02)	0.57	-3.26 (-12.92, 7.48)	0.54
>28	91/3291 (2.8)	3.96 (2.88, 4.97)	-5.33 (-15.2, 5.7)	0.33	-3.22 (-14.5, 9.54)	0.60
Light exercise, hrs/wk						
0-4	1041/3285 (31.7)	4.00 (3.18, 4.93)	1.06 (-3.17, 5.49)	0.63		
5-9	1082/3285 (32.9)	3.95 (3.21, 4.85)	-0.96 (-5.05, 3.29)	0.65		
≥10	1162/3285 (35.4)	3.95 (3.10, 4.84)	Referent			

Predictor	N Seropositive (%)	Median titre, U/ml (IQR)	Minimally Adjusted % difference (95% CI) [1]	P value	Fully Adjusted % difference (95% CI) [2]	P value
Vigorous exercise, hrs/wk						
0	1270/3281 (38.7)	3.98 (3.17, 4.86)	1.3 (-3.19, 6)	0.58		
1-3	1242/3281 (37.9)	3.94 (3.15, 4.85)	-0.57 (-5, 4.07)	0.81		
≥4	769/3281 (23.4)	3.97 (3.11, 4.88)	Referent			
Sleep, hrs/night						
≤5	286/3291 (8.7)	4.07 (3.20, 4.96)	3.06 (-3.72, 10.33)	0.39		
6	821/3291 (24.9)	4.02 (3.22, 4.94)	0.82 (-4, 5.89)	0.74		
7	1375/3291 (41.8)	3.92 (3.12, 4.80)	-2.89 (-7.06, 1.47)	0.19		
≥8	809/3291 (24.6)	3.93 (3.12, 4.88)	Referent			
Self-assessed general health						
Excellent	651/3291 (19.8)	3.95 (3.17, 4.91)	Referent			
Very good	1300/3291 (39.5)	3.93 (3.13, 4.81)	-1.38 (-5.96, 3.42)	0.57		
Good	916/3291 (27.8)	4.00 (3.10, 4.84)	1.19 (-3.82, 6.46)	0.65		
Fair	337/3291 (10.2)	4.02 (3.32, 4.94)	3.71 (-2.96, 10.84)	0.28		
Poor	87/3291 (2.6)	4.07 (2.91, 5.34)	5.04 (-6.18, 17.6)	0.39		
Anxiety or depression						
No	2525/3290 (76.7)	3.94 (3.12, 4.84)	Referent			
Yes	765/3290 (23.3)	4.02 (3.27, 4.93)	1.96 (-2.16, 6.25)	0.36		
Food choice						
None	3122/3291 (94.9)	3.96 (3.15, 4.85)	Referent			
Vegetarian	134/3291 (4.1)	3.93 (3.22, 5.01)	-2.68 (-10.84, 6.23)	0.54	-5.01 (-14.4, 5.41)	0.33
Vegan	35/3291 (1.1)	4.50 (3.42, 5.82)	16.03 (-1.93, 37.28)	0.08	16.23 (-7.2, 45.59)	0.19
Medical conditions						
Heart disease [3]						
No	3120/3291 (94.8)	3.97 (3.15, 4.87)	Referent			
Yes	171/3291 (5.2)	3.87 (3.03, 4.76)	3.81 (-4.11, 12.38)	0.36		
Arterial disease [4]						
No	3063/3291 (93.1)	3.97 (3.15, 4.88)	Referent			
Yes	228/3291 (6.9)	3.87 (3.16, 4.71)	3.87 (-3.09, 11.33)	0.28		
Hypertension						
No	2459/3291 (74.7)	4.01 (3.21, 4.93)	Referent			
Yes	832/3291 (25.3)	3.83 (3.00, 4.70)	-4.92 (-8.75, -0.94)	0.02	-6.14 (-10.83, -1.21)	0.015*
Immunodeficiency [5]						
No	3266/3291 (99.2)	3.97 (3.15, 4.86)	Referent			
Yes	25/3291 (0.8)	3.69 (2.99, 4.44)	0.60 (-17.56, 22.76)	0.95		
Major neurological conditions [6]						
No	3179/3291 (96.6)	3.97 (3.15, 4.86)	Referent			

Predictor	N Seropositive (%)	Median titre, U/ml (IQR)	Minimally Adjusted % difference (95% CI) [1]	P value	Fully Adjusted % difference (95% CI) [2]	P value
Yes	112/3291 (3.4)	3.88 (3.08, 4.82)	4.39 (-5.11, 14.84)	0.38		
Cancer						
Never	2899/3291 (88.1)	3.97 (3.15, 4.85)	Referent			
Past (cured or in remission)	351/3291 (10.7)	3.96 (3.12, 4.93)	1.93 (-3.68, 7.87)	0.51		
Present (active)	41/3291 (1.2)	3.68 (3.25, 4.49)	-3.75 (-17.69, 12.56)	0.63		
Asthma						
No	2793/3291 (84.9)	3.98 (3.16, 4.86)	Referent			
Yes	498/3291 (15.1)	3.88 (3.09, 4.84)	-4.51 (-9.03, 0.24)	0.06	-2.52 (-7.89, 3.16)	0.38
COPD						
No	3206/3291 (97.4)	3.97 (3.16, 4.86)	Referent			
Yes	85/3291 (2.6)	3.75 (2.75, 4.55)	-3.60 (-13.56, 7.50)	0.51		
Diabetes types						
No diabetes	2989/3283 (91.0)	3.98 (3.17, 4.90)	Referent			
Pre-diabetes	99/3283 (3.0)	3.75 (2.78, 4.39)	-5.96 (-15.03, 4.06)	0.23		
Type 1 diabetes	32/3283 (1.0)	3.80 (2.90, 4.27)	-13.25 (-27.25, 3.44)	0.11		
Type 2 diabetes	163/3283 (5.0)	3.86 (3.00, 4.80)	-0.75 (-8.38, 7.51)	0.85		
Atopy [7]						
No	2465/3291 (74.9)	3.99 (3.15, 4.88)	Referent			
Yes	826/3291 (25.1)	3.91 (3.12, 4.82)	-2.23 (-6.06, 1.74)	0.27		
Pre-vaccination SARS-COV-2 infection/COVID-19						
Seronegative	1705/2012 (84.7)	3.91 (3.15, 4.67)	Referent			
Seropositive, asymptomatic	215/2012 (10.7)	4.68 (3.76, 6.77)	37.46 (28.71, 46.8)	<0.001	36.06 (27.33, 45.39)	<0.001*
Seropositive, symptomatic	92/2012 (4.6)	7.29 (4.63, 13.66	82.25 (66.61, 99.35)	<0.001	84.75 (68.42, 102.67)	<0.001*
Nutritional supplements						
Multivitamin						
No	2603/3291 (79.1)	3.94 (3.15, 4.84)	Referent			
Yes	688/3291 (20.9)	4.03 (3.15, 4.93)	0.11 (-4.06, 4.46)	0.96		
Vitamin A						
No	3275/3291 (99.5)	3.96 (3.15, 4.86)	Referent			
Yes	16/3291 (0.5)	3.96 (3.05, 4.96)	8.61 (-15.24, 39.16)	0.51		
Vitamin C						
No	2959/3291 (89.9)	3.96 (3.14, 4.88)	Referent			
Yes	332/3291 (10.1)	4.06 (3.25, 4.79)	1.59 (-4.07, 7.58)	0.59		
Vitamin D						
No	1609/3291 (48.9)	3.98 (3.17, 4.86)	Referent			
Yes	1682/3291 (51.1)	3.94 (3.12, 4.86)	-0.45 (-3.83, 3.04)	0.8		

Predictor	N Seropositive (%)	Median titre, U/ml (IQR)	Minimally Adjusted % difference (95% CI) [1]	P value	Fully Adjusted % difference (95% CI) [2]	P value
Zinc						
No	3118/3291 (94.7)	3.96 (3.15, 4.85)	Referent			
Yes	173/3291 (5.3)	3.94 (3.27, 4.94)	4.68 (-3.11, 13.10)	0.25		
Selenium						
No	3256/3291 (98.9)	3.96 (3.15, 4.85)	Referent			
Yes	35/3291 (1.1)	4.17 (3.41, 5.68)	9.89 (-7.11, 30.00)	0.27		
Iron						
No	3193/3291 (97.0)	3.96 (3.15, 4.86)	Referent			
Yes	98/3291 (3.0)	4.00 (3.23, 4.99)	0.32 (-9.38, 11.07)	0.95		
Probiotics						
No	3091/3291 (93.9)	3.96 (3.15, 4.85)	Referent			
Yes	200/3291 (6.1)	4.01 (3.13, 5.16)	4.62 (-2.67, 12.45)	0.22		
Omega-3						
No	2874/3291 (87.3)	3.96 (3.15, 4.84)	Referent			
Yes	417/3291 (12.7)	4.00 (3.21, 5.00)	3.56 (-1.68, 9.08)	0.19		
Cod liver oil						
No	2987/3291 (90.8)	3.96 (3.14, 4.86)	Referent			
Yes	304/3291 (9.2)	4.03 (3.21, 4.88)	6.24 (0.05, 12.80)	0.05	4.18 (-2.96, 11.84)	0.26
Garlic						
No	3231/3291 (98.2)	3.96 (3.15, 4.86)	Referent			
Yes	60/3291 (1.8)	4.24 (3.42, 5.29)	10.80 (-2.62, 26.07)	0.12		
Medications						
Beta-2 adrenergic agonists						
No	2993/3291 (90.9)	3.98 (3.16, 4.86)	Referent			
Yes	298/3291 (9.1)	3.80 (3.06, 4.77)	-4.31 (-9.90, 1.63)	0.15		
Beta blockers						
No	3007/3291 (91.4)	3.97 (3.16, 4.86)	Referent			
Yes	284/3291 (8.6)	3.82 (3.02, 4.85)	0.47 (-5.59, 6.93)	0.88		
Statins						
No	2547/3291 (77.4)	4.03 (3.20, 4.96)	Referent			
Yes	744/3291 (22.6)	3.74 (3.02, 4.61)	-3.33 (-7.52, 1.05)	0.13		
ACE inhibitors						
No	2896/3291 (88.0)	3.97 (3.17, 4.88)	Referent			
Yes	395/3291 (12.0)	3.89 (2.99, 4.74)	-0.55 (-5.76, 4.95)	0.84		
Proton pump inhibitors						
No	2742/3291 (83.3)	3.98 (3.15, 4.88)	Referent			
Yes	549/3291 (16.7)	3.89 (3.13, 4.77)	1.71 (-2.92, 6.56)	0.48		

Predictor	N Seropositive (%)	Median titre, U/ml (IQR)	Minimally Adjusted % difference (95% CI) [1]	P value	Fully Adjusted % difference (95% CI) [2]	P value
H2-receptor antagonists						
No	3268/3291 (99.3)	3.96 (3.15, 4.86)	Referent			
Yes	23/3291 (0.7)	4.04 (3.27, 4.84)	-0.59 (-19.18, 22.28)	0.96		
Inhaled corticosteroids						
No	3069/3291 (93.3)	3.96 (3.15, 4.86)	Referent			
Yes	222/3291 (6.7)	3.96 (3.30, 4.90)	-0.78 (-7.38, 6.29)	0.82		
Bronchodilators						
No	2977/3291 (90.5)	3.98 (3.15, 4.86)	Referent			
Yes	314/3291 (9.5)	3.84 (3.06, 4.88)	-2.42 (-7.99, 3.49)	0.41		
Systemic Immunosuppressants						
No	3118/3291 (94.7)	3.98 (3.18, 4.87)	Referent			
Yes	173/3291 (5.3)	3.61 (2.53, 4.75)	-11.62 (-18.18, -4.53)	0.002	-8.81 (-17.39, 0.66)	0.07
Angiotensin receptor blockers						
No	3078/3291 (93.5)	3.98 (3.17, 4.90)	Referent			
Yes	213/3291 (6.5)	3.69 (2.94, 4.43)	-7.46 (-13.79, -0.66)	0.03	-0.24 (-8.76, 9.08)	0.96
Selective serotonin reuptake inhibitors						
No	3079/3291 (93.6)	3.96 (3.14, 4.85)	Referent			
Yes	212/3291 (6.4)	4.04 (3.32, 4.99)	1.56 (-5.38, 9.01)	0.67		
non-SSRIs antidepressants						
No	3156/3291 (95.9)	3.97 (3.15, 4.86)	Referent			
Yes	135/3291 (4.1)	3.77 (3.21, 4.93)	1.69 (-6.79, 10.94)	0.71		
Calcium channel blockers						
No	2916/3291 (88.6)	3.97 (3.16, 4.90)	Referent			
Yes	375/3291 (11.4)	3.89 (3.04, 4.72)	0.24 (-5.15, 5.94)	0.93		
Thiazides						
No	3171/3291 (96.4)	3.97 (3.15, 4.86)	Referent			
Yes	120/3291 (3.6)	3.80 (3.11, 4.90)	-2.09 (-10.73, 7.39)	0.65		
Vitamin K antagonists						
No	3258/3291 (99.0)	3.97 (3.15, 4.87)	Referent			
Yes	33/3291 (1.0)	3.31 (2.78, 3.98)	-15.22 (-28.71, 0.82)	0.06	-17.15 (-35.51, 6.43)	0.14
Sodium-glucose co-transporter-2 inhibitors						
No	3271/3291 (99.4)	3.96 (3.15, 4.86)	Referent			
Yes	20/3291 (0.6)	4.38 (3.93, 5.10)	13.83 (-8.87, 42.18)	0.25		
Anticholinergics						
No	3110/3291 (94.5)	3.97 (3.15, 4.86)	Referent			
Yes	181/3291 (5.5)	3.81 (3.11, 4.80)	-2.09 (-9.23, 5.61)	0.58		
Metformin						

Predictor	N Seropositive (%)	Median titre, U/ml (IQR)	Minimally Adjusted % difference (95% CI) [1]	P value	Fully Adjusted % difference (95% CI) [2]	P value
No	3173/3291 (96.4)	3.96 (3.15, 4.86)	Referent			
Yes	118/3291 (3.6)	4.04 (3.12, 4.86)	3.50 (-5.71, 13.61)	0.47		
Bisphosphonates						
No	3219/3291 (97.8)	3.97 (3.15, 4.86)	Referent			
Yes	72/3291 (2.2)	3.60 (2.48, 4.90)	-6.75 (-17.15, 4.96)	0.25		
Anti-platelet drugs						
No	3024/3291 (91.9)	3.98 (3.17, 4.88)	Referent			
Yes	267/3291 (8.1)	3.82 (3.02, 4.75)	2.46 (-3.99, 9.34)	0.46		
Sex hormone therapy						
No	3034/3291 (92.2)	3.96 (3.13, 4.85)	Referent			
Yes	257/3291 (7.8)	4.06 (3.29, 5.22)	0.99 (-5.39, 7.81)	0.77		
Aspirin [8]						
No	3084/3291 (93.7)	3.97 (3.17, 4.87)	Referent			
Yes	207/3291 (6.3)	3.89 (2.89, 4.77)	1.48 (-5.62, 9.11)	0.69		
Paracetamol [8]						
No	3121/3291 (94.8)	3.97 (3.15, 4.86)	Referent			
Yes	170/3291 (5.2)	3.83 (3.05, 4.83)	2.04 (-5.62, 10.32)	0.61		
BCG vaccinated						
No	399/3283 (12.2)	3.89 (3.00, 4.90)	Referent			
Yes	2601/3283 (79.2)	3.99 (3.20, 4.89)	3.48 (-1.89, 9.15)	0.8		

Abbreviations: IQR, inter quartile range; CI, confidence interval; BMI, body mass index; IMD, index of multiple deprivation; COPD, chronic obstructive pulmonary disease; SARS-COV-2, severe acute respiratory syndrome coronavirus 2; COVID-19, coronavirus disease 2019; ACE, angiotensin-converting enzyme; H2, histamine 2; SSRIs, selective serotonin reuptake inhibitors; BCG, bacillus calmette-guérin

(1) Adjusted for age and sex only
(2) Adjusted for age, sex, time since fully vaccinated, inter-dose interval, time of second vaccination, quarter of second vaccination, ethnicity, quantiles of IMD, alcohol consumption, food choice, hypertension, asthma, pre-vaccination SARS-CoV-2 status, cod liver oil, systemic immunosuppressants, selective serotonin reuptake inhibitors, angiotensin receptor blockers, and vitamin K antagonists
3) Heart disease defined as coronary artery disease or heart failure
(4) Arterial disease defined as ischaemic heart disease, peripheral vascular disease or cerebrovascular disease
(5) Immunodeficiency defined as HIV, primary immune deficiency or other immunodeficiency
(6) Major neurological conditions defined as stroke, transient ischaemic attack, dementia, Parkinson's disease, multiple sclerosis or motor neuron disease
(7) Atopy defined as atopic eczema/dermatitis and/or hayfever/allergic rhinitis
(8) Chronic use prior to vaccination (i.e. distinct from acute post-vaccination use for treatment of reactogenic symptoms)

* Below multiple comparisons testing critical p-value threshold of 0.031

Table S5. Influence of post-vaccination use of paracetamol/NSAIDs on antibody titres

Predictor		Median IgGAM ratio (IQR)	Adjusted for main model predictors,[1] % difference (95% CI)	P value	Additionally adjusted for presence vs. absence of post-vaccination symptoms,[2] % difference (95% CI)	P value
**Use of post-vaccination paracetamol / NSAIDs**	None	2.91 (1.93, 4.09)	Referent		Referent	
	After 1st or 2nd doses	2.75 (1.85, 3.93)	3.38 (0.25, 6.60)	0.034	0.67 (-2.27, 3.69)	0.66
	After both doses	2.95 (1.97, 4.28)	5.86 (2.22, 9.62)	0.001	2.98 (-0.40, 6.49)	0.09

Abbreviations: IQR, inter-quartile range; CI, confidence interval ; NSAID, non-steroidal anti-inflammatory drug

(1) Main model predictors: vaccine type, time from 2nd vaccine dose to sampling, inter-dose interval, quarter of 2nd vaccine dose, age, ethnicity, BMI, hypertension and pre-vaccination COVID-19 status

(2) Post-vaccination symptoms include any of the following: tenderness/soreness at the injection site, headache, fever or muscle aches

Supplementary Figures

Figure S1. Study profile

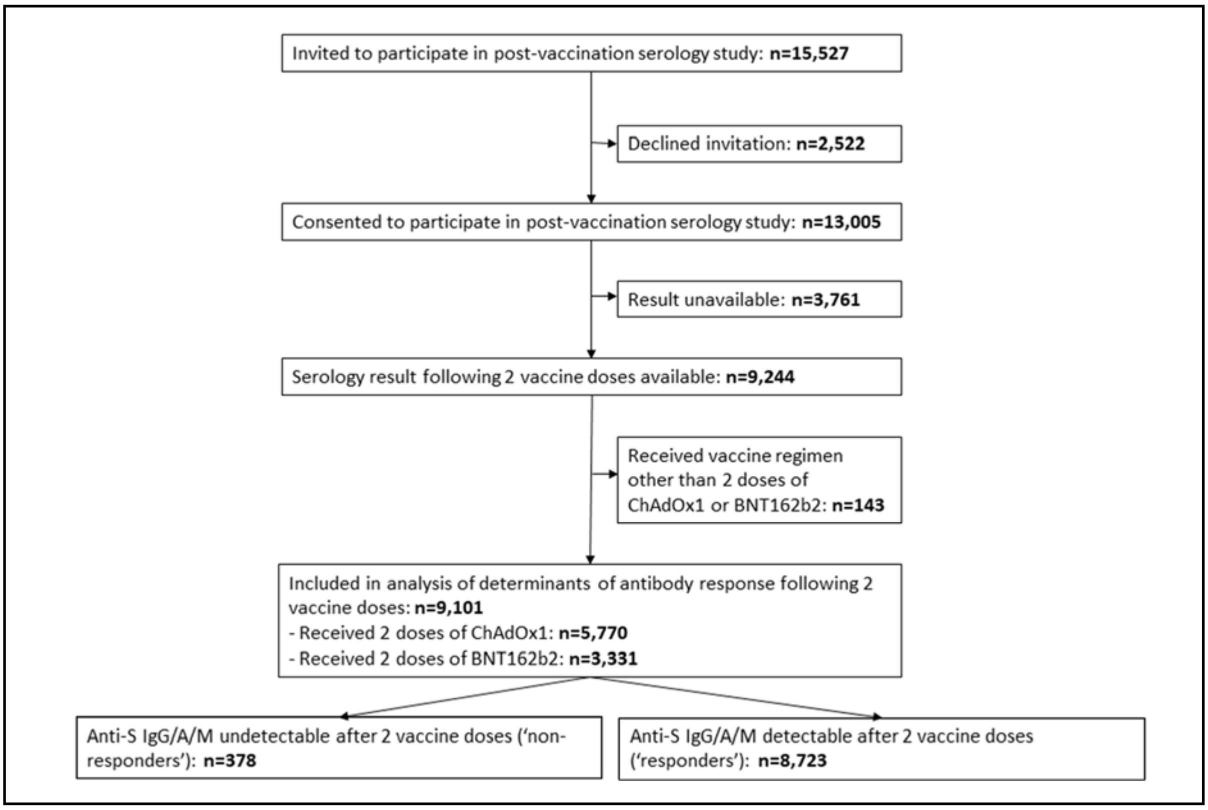

Figure S2. Post-vaccination SARS-CoV-2 antibody titres by vaccine type vs. diagnosis of hypertension (A), prescribed immunosuppressant medications (B) or consumption of selenium supplements (C).

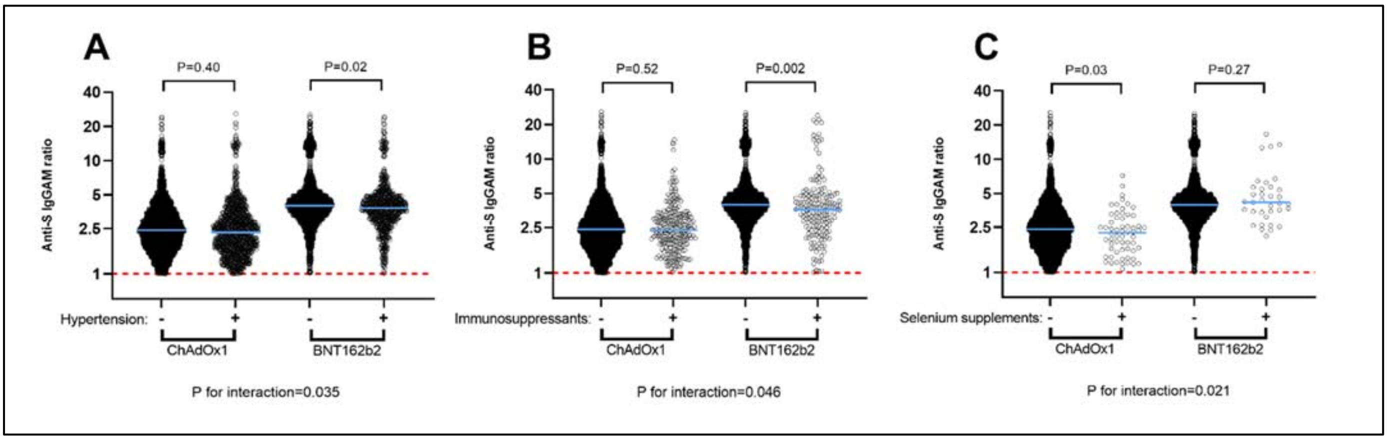

Supplement: Supplementary file 1 [file vaccines-10-01601-s001.zip › vaccines-1904951-supplementary.pdf]
